# Grape Must as a Bioelectrochemical Processor

**DOI:** 10.1021/acsomega.5c10998

**Published:** 2026-02-17

**Authors:** Panagiotis Mougkogiannis, Andrew Adamatzky

**Affiliations:** Unconventional Computing Laboratory, 1981 University of the West of England, Bristol BS16 1QY, U.K.

## Abstract

We explore spontaneous voltage oscillations in grape must (mustalevria) fermentation systems. This study uses multichannel differential electrode arrays. Seven platinum–iridium (Pt/Ir) electrode pairs tracked bioelectrochemical changes for 200,000 s. They showed complex patterns over time and space. Frequencies varied from 0.00044 to 0.00215 Hz. Power spectral density analysis showed brown noise traits. The spectral slopes ranged from −2.01 to −3.28. This indicates strong temporal integration and memory effects during fermentation. Environmental correlation analysis showed temperature as the primary modulator (*r* = 0.245–0.558), while humidity exhibited negative correlations (−0.052 to −0.245). Binary state analysis showed that the system uses natural Boolean logic. XOR gates had the highest entropy at 0.93 bits. This suggests that there is significant temporal asynchrony across different spatial areas. Principal component analysis found activation patterns without a single strong mode. It needed 3–4 components to capture 77.6% of the system’s variance. The fermentation medium showed uneven metabolic activity across different areas. Also, the electrode locations were statistically independent, with mutual information below 0.206 bits. These findings show that traditional food fermentation systems work like self-organizing bioelectrochemical processors. They can also perform distributed computation through local metabolic interactions. Brown noise scaling and memory effects can impact fermentation monitoring and control. This means short-term measurements may not accurately predict long-term behavior. This work shows that grape must fermentation can be a model system. It helps us study new computational properties in biological electrochemical systems.

## Graphical abstract

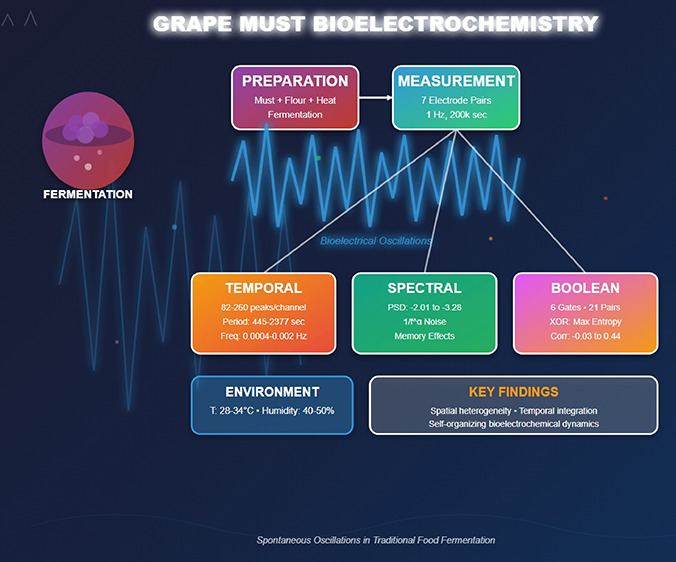

## Introduction

Biological fermentation is key in food science and industrial biotechnology. It relies on complex, self-organizing microbial ecosystems.^1^ These systems create constant, spontaneous bioelectrochemical activity.^2–6^ This happens alongside the visible effects of gas production and substrate use.^7,8^ This activity comes from closely linked physical and biological events. It includes localized ionic transport across cell membranes,^9^ redox reactions driven by metabolic intermediates, and coordinated microbial physiological changes.^10^ These electrical dynamics are everywhere, but we still do not fully understand how they work or are organized over time and space. This is especially true in traditional food fermentation, where local microbial communities are kept intact. A big knowledge gap remains about how these electrical signals affect, or are affected by, the overall metabolic state of the fermentation system. Understanding the relationship between electrical signals and metabolic state is critical for three reasons. First, electrical measurements offer noninvasive, continuous monitoring that could enable real-time fermentation control without destructive sampling—a capability particularly valuable for traditional fermentations where maintaining microbial ecology is essential. Second, bioelectrochemical signals may encode information about spatial metabolic organization that bulk chemical measurements cannot capture, potentially revealing how microbial communities self-organize in natural fermentation systems. Third, if electrical dynamics reflect metabolic processes, they could serve as early indicators of fermentation state transitions, enabling predictive control strategies that anticipate rather than react to process changes. However, these applications require first establishing that spontaneous voltage oscillations exist in traditional fermentation systems, characterizing their spatiotemporal properties, and demonstrating their sensitivity to biological and environmental factors—prerequisites that remain unmet in current literature.

Previous studies of fermentation have focused overwhelmingly on chemical and microbiological characterization—measuring metabolite concentrations, microbial populations, and enzymatic activities—while treating electrical phenomena as secondary effects or ignoring them entirely.^11–15^ This biochemical-centric view misses a potentially rich source of information: bioelectrochemical signals arise from the integrated activity of ionic transport, redox reactions, and membrane potentials across entire microbial populations, potentially encoding collective behaviors invisible to single-cell or bulk chemical measurements. Moreover, electrical signals propagate rapidly through fermentation media via ionic conduction and potentially through microbial electrical coupling, suggesting they may mediate spatial coordination among distant regions—a possibility unexplored in traditional fermentation research.

To fill this gap, we need a system that effectively shows complex, natural microbial-driven bioelectrochemistry. Grape must, or mustalevria, is a great example of an ecologically rich system.^16–18^ Its complexity comes from a dense, nutrient-rich carbohydrate matrix and a strong, native yeast and bacteria population.^19^ This is often kept intact using traditional preparation methods. This context helps include all types of native microbes.^20^ This allows us to see strong, self-organizing electrical behaviors that might not show up in simpler lab media. To resolve these spatial dynamics, advanced measurement approaches are essential.

Traditional analytical methods, like bulk electrochemical measurements and single-point voltage monitoring, hide the important spatial differences needed to understand complex biological groups. While multichannel electrode arrays are well-established in fields such as neuroscience and electrophysiology,^21,22^ their application to characterizing spatial heterogeneity in traditional food fermentation systems has not been previously reported. This study uses a new method with multichannel electrode arrays. It specifically involves seven pairs of differential Platinum/Iridium (Pt/Ir) electrodes. The 10 mm electrode spacing was selected based on preliminary experiments indicating that metabolic gradients in grape must fermentation develop at centimeter scales, reflecting substrate diffusion limitations and spatial organization of microbial colonies.^23^ While this spacing is coarse compared to high-density neural recording arrays, it is appropriate for resolving the characteristic length scales of fermentation dynamics. This setup allowed for spatially resolved monitoring of voltage changes in different locations. While temperature and humidity are known to affect enzymatic kinetics and fermentation rates, their influence on spontaneous bioelectrochemical oscillations remains poorly characterized—a gap this work begins to address.

To go beyond detecting signals and understand the meaning of these complex electrical patterns, we use advanced information-theoretic methods.^24,25^ Information-theoretic methods are great for analyzing multichannel bioelectrochemical data. They measure relationships between different spatial measurements. Plus, they do not need assumptions about underlying mechanisms or signal linearity. Information theory, like mutual information and Shannon entropy, goes beyond traditional correlation analysis. It finds all types of statistical dependencies, even nonlinear ones. Boolean logic analysis and state space characterization show how multiple channels work together. This is different from spectral methods that focus on individual channels. These approaches help us understand system-level patterns better. These methods are common in neuroscience for analyzing multielectrode recordings. They are also used in complex systems research to study self-organization. So, they are great tools for exploring if fermentation shows similar collective dynamics. Information-theoretic metrics offer a way to measure system organization without relying on specific dimensions. This means we can compare different biological systems and their spatial scales easily.

The analysis uses techniques like natural Boolean logic operations and Principal Component Analysis (PCA), which help to interpret the computational properties and group dynamics in the multivariate voltage data.^26^ This method shifts how we see fermentation systems. They become a distributed, self-programming bioreactor that does unique computations.^27^ This creates a new connection between biological electrochemistry and information science. The observed scaling matches complex biological events. These often show fractal and nonlinear dynamics.^28,29^

We hypothesize the voltage changes come from linked metabolic and electrochemical feedback loops. These loops work across different spatial and temporal scales. They create collective behaviors like self-organized criticality in complex biological systems. We employ spectral analysis to examine frequency characteristics and temporal scaling properties,^30,31^ information-theoretic methods to quantify interchannel relationships, and environmental correlation analysis to assess external modulation of bioelectrochemical dynamics. Our approach aims to determine whether traditional fermentation systems exhibit spatially organized electrical activity and, if so, whether such activity displays signatures of collective microbial behavior documented in other biological systems.^32–36^ The findings may inform both fundamental understanding of microbial electrochemistry and practical applications in fermentation monitoring and bioinspired computing.

This study focuses on characterizing the spatiotemporal properties of spontaneous voltage oscillations and their information-theoretic features. We intentionally employed a noninvasive, purely electrical measurement approach to avoid disturbing the traditional fermentation process. Consequently, we do not provide direct parallel measurements of metabolic parameters (pH, dissolved oxygen, metabolite concentrations, microbial density) that would enable quantitative correlation between electrical signals and specific biochemical processes. This represents a limitation of the current work, and we frame our metabolic interpretations as hypotheses consistent with the observed electrical dynamics rather than definitively established mechanisms. Future studies with integrated multimodal sensing are needed to validate these interpretations.

## Experimental Methods

### Preparation of Mustalevria

The grape grape must in this study came from Roditis (*V. itisvinifera*), a classic Greek white wine grape. It was harvested at the end of August 2025 from vineyards in the Patras region of northern Peloponnese, Greece. Roditis is well-suited for producing traditional *mustalevria*. It has a balanced sugar content of 18°–20° Brix at harvest and a moderate acidity of pH 3.2–3.4. The grapes were picked at optimal ripeness, based on refractometric measurements and organoleptic (taste) assessments. This variety also hosts a strong population of native yeasts, making it ideal for studying spontaneous fermentation without the need for commercial inoculation.^37,38^

Fresh grape grape must was obtained by crushing and pressing whole Roditis grape clusters within 4 h of harvest to preserve the native microbial consortium. The grape must contained approximately 180–200 g/L of fermentable sugars, primarily glucose and fructose. It also had 5–7 g/L of titratable acidity (expressed as tartaric acid) and indigenous yeast populations in the range of 10^4^–10^5^ CFU/mL, consistent with earlier studies of Greek grape varieties.^39^

To prepare the mustalevria, seven tablespoons of wheat flour per liter of fresh grape must were gradually incorporated while stirring to prevent lump formation. This flour addition (approximately 70 g/L) serves two purposes: it adds fermentable carbohydrates and gives mustalevria its characteristic thick texture. The mixture was then subjected to controlled thermal processing at 75–80 °C for 15 min with continuous stirring. This temperature range partially gelatinizes the starch while preserving heat-tolerant components of the native microbiota, including *Saccharomyces cerevisiae* strains adapted to the Roditis grape ecosystem.

After heating, the mixture was transferred into a ceramic vessel (30 cm wide, 15 cm deep) and allowed to cool to room temperature (approximately 25 °C). During cooling, a surface pellicle began to form within 2–3 h, indicating the onset of aerobic microbial activity. The relatively high pH of Roditis grape must facilitates the emergence of a diverse microbial community, contributing to the complex bioelectrochemical patterns observed during fermentation.

The thermal processing phase includes careful heating and constant stirring. This helps partially gelatinize the flour starch. It also keeps the beneficial microorganisms in the raw grape must alive. If flour sticks, it can cause hot spots and uneven texture. Surface bubbling shows that starch granules are swelling. They release amylose and amylopectin into the water. This creates the viscosity needed for fermentation and gelation. After removing it from heat, we pour the mixture into serving containers. This starts a complex cooling phase with many biochemical changes. As the temperature drops, leftover enzymes from the grape must and flour change the carbohydrate matrix. At the same time, native yeasts and bacteria start forming metabolic pathways. These pathways create the bioelectrochemical oscillations seen during fermentation. A thin layer forms during cooling, like the cellulosic pellicle in kombucha SCOBY.^40^ This layer shows that aerobic microbes are developing. These microbes help the fermentation ecosystem and may affect where electrochemical activity occurs, as seen in differential electrode arrays.

### Electrode Array Configuration and Positioning Control

The electrodes were inserted vertically into the fermentation medium to a controlled depth of approximately 5–7 cm (roughly half the vessel depth of 15 cm), ensuring they remained fully submerged throughout the measurement period while avoiding contact with the vessel bottom (Figure 1). The 10 mm separation between electrode pairs was maintained using prefabricated rigid holders. Spatial positioning of different electrode pairs was arranged to sample distinct horizontal regions of the vessel (radius ∼ 15 cm), with typical interpair distances of 3–5 cm. The 10 mm electrode pair separation was chosen based on several considerations: (1) the characteristic diffusion length scale for oxygen and metabolites in fermentation media over the time scale of our observed oscillations (400–2300 s) is estimated at 1–10 mm using *D* ≈ 10^–6^ cm^2^/s and $L∼\sqrt{Dt}$ ; (2) yeast colony dimensions and microenvironmental zones typically span millimeter to centimeter scales; and (3) our results validate this choice—the statistical independence between channels (mutual information <0.206 bits) demonstrates that the electrode spacing effectively samples distinct metabolic microenvironments rather than redundantly measuring the same process.^41^ Regarding how different spacings would affect results: Smaller separation (1–5 mm) would likely show higher interelectrode correlations and mutual information (channels would sample overlapping microenvironments), potentially revealing finer-scale heterogeneity but with reduced spatial independence—we might observe more coordinated oscillations and lower XOR entropy as adjacent measurements track the same local processes. Larger separation (20–50 mm) would likely show even lower correlations approaching our current values (already quite low at <0.45), potentially missing coordinated patterns entirely if electrodes sample completely independent regions—we might observe similar or even lower mutual information but lose information about spatial coupling mechanisms. Our subsequent analysis validates this choice: the weak-to-moderate correlations (mostly <0.45, Figure 6) demonstrate that electrode positions sample genuinely independent microenvironments rather than redundantly measuring the same process.

**Figure 1:**
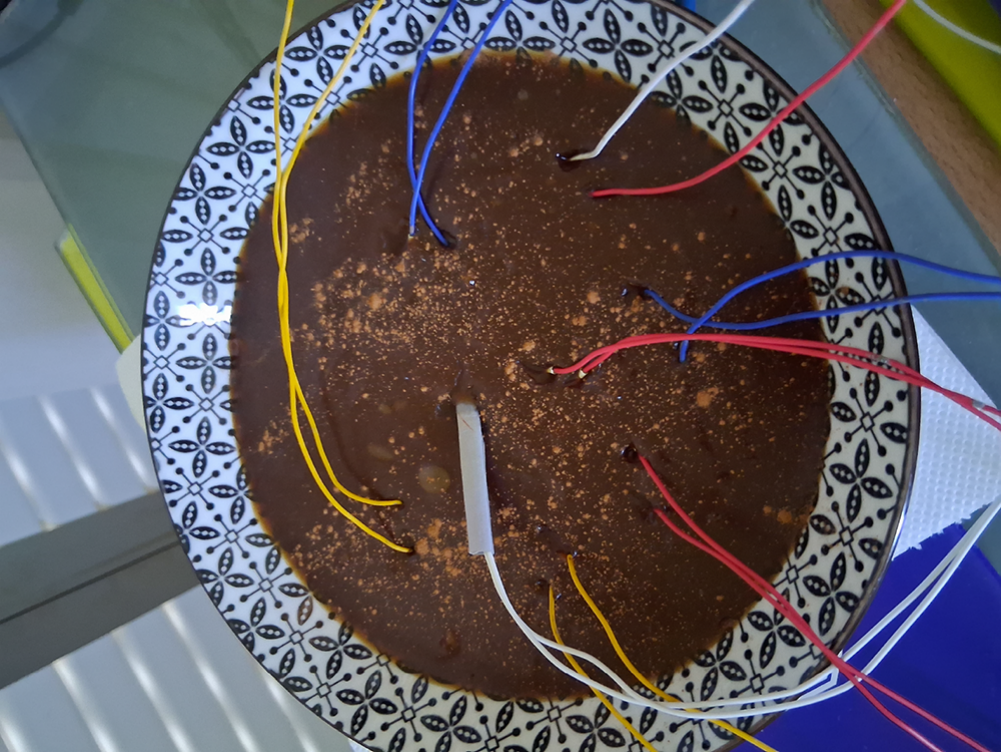
Experimental setup for spontaneous voltage oscillation measurements in grape must (mustalevria) fermentation system. Multiple Pt/Ir electrode pairs (10 mm spacing) are inserted into the fermentation medium within a ceramic vessel. Color-coded wires connect electrodes to an ADC-24 data logger for multichannel differential voltage monitoring at 1 Hz sampling rate. The dark brown fermentation mixture contains visible grape particles and sediments.

We used a Pico Technology ADC-24 data logger with built-in differential amplification and 24-bit resolution. The system includes hardware antialiasing filters with a cutoff frequency of 0.4 Hz (below our 1 Hz sampling rate). No additional external amplification was applied. Software filtering was not performed on the raw data to preserve all frequency components for spectral analysis. Each measurement channel represents the differential voltage between two Pt/Ir electrodes (10 mm separation) within the fermentation medium, without a common reference electrode. While we acknowledge that Pt/Ir electrodes are polarizable and this configuration is susceptible to drift, our control experiments (described below) demonstrate that this does not compromise our primary findings regarding oscillatory dynamics.

We performed extensive control experiments during our study. Prefermentation measurements in sterile grape must (heat-treated at 121 °C for 20 min to eliminate viable microorganisms) showed baseline drift (<2 mV over 24 h) but completely lacked the periodic oscillations observed in active fermentation. We systematically compared raw signals versus detrended signals. Drift occurs at very low frequencies (<0.0001 Hz, periods >10,000 s), whereas the metabolic oscillations we analyze occupy a distinct frequency band (0.0004–0.002 Hz, periods 400–2300 s). Our analyses demonstrate clear separation between these phenomena. Postexperiment measurements in pH 3.5 tartaric acid buffer (mimicking grape must acidity) at constant temperature characterized electrode drift rates of 0.5–1.5 mV/hour. Critically, these buffer measurements showed monotonic drift without oscillatory features, confirming that the oscillations arise from biological activity. The seven-channel configuration was determined by available data acquisition hardware (ADC-24 with eight differential inputs) rather than theoretical considerations about optimal spatial sampling density. More channels would provide finer spatial resolution and potentially reveal additional organizational scales, while fewer channels would reduce spatial information. The relationship between electrode array size and captured system complexity remains an open question for future parametric studies.

## Results and Discussion

### Peak Detection Analysis in Grape Must Fermentation Systems

#### Mathematical Framework for Peak Identification

The automated peak detection algorithm in this study uses a threshold-based method^42^ and includes a minimum-distance rule to identify significant voltage changes in the multichannel electrode data. The peak detection criterion is mathematically defined as

$$P_{i}=\left\{t_{i}:V\left(t_{i}\right)>V_{\mathrm{threshold}}\wedge \underset{j\neq i}{\min}|t_{i}-t_{j}|>\Delta t_{\min}\right\}$$ 1

Here, *P*_*i*_ represents the set of detected peaks, and *V*(*t*_*i*_) is the voltage at time *t*_*i*_. The amplitude threshold is *V*_threshold_ = 4.0 mV. The minimum time gap between consecutive peaks is Δ*t*_min_ = 400 samples (equivalent to 400 s at a 1 Hz sampling rate).

The algorithm further incorporates a prominence filter to ensure that detected peaks represent genuine oscillatory events rather than noise artifacts:

$$\mathrm{Prominence}\left(t_{i}\right)=\min\left(V\left(t_{i}\right)-V_{\mathrm{left}},V\left(t_{i}\right)-V_{\mathrm{right}}\right)$$ 2

where *V*_left_ and *V*_right_ denote the lowest voltage values to the left and right of the peak *t*_*i*_ within the minimum-distance window.^43^ This mathematical framework ensures robust identification of physiologically relevant oscillations while minimizing false positives due to baseline fluctuations and instrumental noise. We employed prominence filtering rather than frequency-domain filtering or baseline subtraction to preserve oscillation waveform characteristics while rejecting baseline drift artifacts. Alternative peak detection methods (wavelet-based, matched filtering, or machine learning approaches) might identify additional or different events; however, our approach prioritizes reproducibility and interpretability over maximum sensitivity, accepting that we may miss low-amplitude events while ensuring high confidence in detected peaks. For each channel, peak times were first identified from the raw voltage trace using a fixed amplitude threshold (4 mV) and a minimum separation of 400 samples between consecutive peaks to avoid counting small local fluctuations. Let *t*_*i*_ denote the time (in seconds) of the *i*th detected peak. The instantaneous oscillation periods were then computed as the temporal differences between successive peaks, Δ*t*_*i*_ = *t*_*i*+1_ – *t*_*i*_. We emphasize that these detected peaks do not represent strictly periodic oscillations; rather, they reflect irregular, transient metabolic events. Consequently, the “average frequency” reported in Table 1 should be interpreted as a characteristic event rate rather than a true oscillation frequency. This metric is calculated as ⟨*f*⟩ = (*N* – 1)/*T*_total_, where *N* is the total number of detected peaks and *T*_total_ is the measurement duration (198,066 s), providing the mean rate at which voltage excursions exceed the detection threshold. The substantial period standard deviations (Table 1)—ranging from 152.78 to 11366.26 s—confirm the highly irregular, nonperiodic nature of these events.

**Table 1:** Peak Detection Summary for Multichannel Grape Must Fermentation Voltage Excursions^a^ ^,^ ^b^

**channel**	**num peaks**	**avg period (s)**	**avg freq (Hz)**	**amplitude STD (mV)**	**period STD (s)**
Channel_1	82	464.74	0.00215	3.95	152.78
Channel_2	38	600.70	0.00166	3.78	266.09
Channel_3	59	1755.60	0.00057	4.83	6046.86
Channel_4	52	851.27	0.00117	5.97	1898.25
Channel_5	260	762.12	0.00131	4.00	490.77
Channel_6	73	1458.86	0.00069	4.69	5340.38
Channel_7	44	2270.65	0.00044	4.90	11366.26

a Automated peak detection performed using a 4.0 mV threshold and a minimum 400-sample distance criterion on seven differential electrode channels sampled at 1 Hz over a 200,000 s measurement period. The “Avg Freq” values represent mean event rates (peaks per unit time) rather than frequencies of periodic oscillations; large period standard deviations confirm the irregular, non-periodic nature of these events. For true frequency-domain characterization, see PSD analysis (Figure 4).

b Note: “Avg Freq” represents the mean rate of threshold-crossing events (peaks per unit time), not the frequency of a periodic oscillation. Large period standard deviations indicate highly irregular, nonperiodic event timing.

For rigorous frequency-domain characterization of the underlying dynamics, we rely on power spectral density (PSD) analysis (Figure 4), which does not assume periodicity and properly characterizes the full spectrum of temporal fluctuations including both coherent oscillations and stochastic components. The average period reported in the Table 1 corresponds to the arithmetic mean of these interpeak intervals, i.e., $\left\langle T\right\rangle =\frac{1}{N-1}\overset{N-1}{\underset{i=1}{\sum }}\left(t_{i+1}-t_{i}\right)$ for *N* detected peaks in that channel. The average frequency was obtained as the inverse of this mean period, ⟨*f*⟩ = 1/⟨*T*⟩.

The minimum temporal separation of Δ*t*_min_ = 400 s was selected based on preliminary analysis of oscillation periods across all channels (Table 1), which revealed characteristic time scales ranging from 465 to 2271 s. This threshold ensures that consecutive peaks represent distinct oscillation cycles rather than substructure within a single event, while remaining sensitive to the fastest observed metabolic dynamics. The choice balances two competing requirements: (1) avoiding false positives from high-frequency noise (requiring longer Δ*t*_min_), and (2) capturing genuine rapid oscillations in metabolically active regions (requiring shorter Δ*t*_min_). The 400 s value corresponds to approximately 85% of the shortest mean period observed (Channel 1:464.74 s), providing adequate separation while preserving physiologically relevant events.

#### Temporal Dynamics and Channel-Specific Oscillatory Patterns

The multichannel analysis shows different patterns over time across the seven electrode channels. You can see this in Figure 3 and find details in Table 1. Channel 1 shows the fastest oscillatory behavior. It has 82 peaks, mostly during the first fermentation phase. The average gap between peaks is 464.74 s, giving a frequency of 0.00215 Hz. Channel 1 shows a burst-like pattern. Most activity happens in the first 50,000 s. After that, it gradually drops to baseline levels. This pattern shows quick use of available materials. Channel 7 shows the longest average oscillation periods at 2270.65 s, or about 37.8 min. It has the lowest frequency at 0.00044 Hz and exhibits sparse oscillatory activity with only 44 detected peaks over 200,000 s—far fewer than other channels. The very large period standard deviation of 11366.26 s reflects the highly variable and infrequent nature of these events rather than regularly spaced oscillations. In contrast, Channel 5 exhibits the most irregular oscillatory behavior, with 260 peaks showing substantial period variability (standard deviation 490.77 s) and continuous switching throughout the measurement period. Channel 7’s sparse activity might be caused by limited substrate availability or unfavorable local conditions at that electrode location, while Channel 5's persistent but variable oscillations suggest a metabolically active region with fluctuating dynamics.

#### Interpretation of Event Rates versus True Oscillation Frequencies

The peak detection analysis quantifies the rate of threshold-crossing events rather than identifying periodic oscillations. This distinction is critical: while Table 1 reports “average frequencies” ranging from 0.00044 to 0.00215 Hz, these values represent mean event rates (number of peaks per unit time) rather than frequencies of periodic signals. The large period standard deviations—for instance, Channel 7’s period variability of 11366.26 s compared to its mean period of 2270.65 s (CV = 5.0)—demonstrate that voltage excursions occur at highly irregular intervals characteristic of stochastic biological processes rather than deterministic oscillators.

We retain this event-rate metric because it provides a useful comparative measure across channels and reflects the characteristic time scale of metabolic activity: Channel 1’s high event rate (0.00215 Hz, one event every 8 min) indicates frequent metabolic bursts, while Channel 7’s low rate (0.00044 Hz, one event every 38 min) suggests sparse, intermittent activity. However, these values should not be interpreted as implying sinusoidal oscillations at the stated frequencies.

The true frequency structure of fermentation dynamics is revealed by power spectral density analysis (Figure 4), which shows broad-spectrum brown noise characteristics (*S*(*f*) ∝ 1/*f* ^γ^ with γ = 2.01–3.28) rather than discrete spectral peaks that would indicate periodic oscillations. This confirms that fermentation dynamics arise from integrated stochastic processes rather than coherent oscillators, consistent with the irregular peak timing observed in time-domain analysis.

#### Peak Morphology and Oscillation Heterogeneity

The detected peaks exhibit substantial morphological variability across channels, reflecting different underlying metabolic processes. Channels 1, 2, and 5 display sharp peaks with rapid rise and decay, characterized by a full width at half-maximum (fwhm) of approximately 200–400 s. This behavior is consistent with transient metabolic events, such as bursts of glycolytic activity or coordinated responses within yeast populations. In contrast, Channels 3, 6, and 7 exhibit broader peaks (fwhm ∼ 800–1500 s) accompanied by more gradual voltage variations. This suggests the presence of sustained metabolic processes or overlapping oscillatory components operating at slightly different frequencies within the electrode sensing region. This morphological diversity has important implications for peak detection. Broad peaks may correspond to compound events arising from the superposition of multiple metabolic oscillations, whereas narrow peaks typically indicate isolated and localized metabolic bursts. The imposed minimum peak separation of 400 s effectively resolves narrow peaks but may occasionally merge closely spaced events occurring within broader peak structures. This conservative criterion prioritizes detection reliability, trading sensitivity for higher confidence. As a result, some short-lived subevents may be missed; however, the detected peaks are more likely to represent genuine metabolic transitions rather than noise or stochastic fluctuations. The observed variation in peak width likely reflects spatial heterogeneity in several key factors, including substrate availability, yeast population density, oxygen gradients, and local buffering capacity. Regions enriched in substrates may sustain prolonged metabolic activity, leading to broader peaks, while higher cell densities can produce sharper, more synchronized oscillations. Differences between aerobic and microaerobic metabolism operate on distinct time scales, and well-buffered microenvironments tend to exhibit smoother and more stable voltage dynamics. Future studies combining electrical measurements with spatially resolved metabolite profiling could elucidate these structure–function relationships in greater detail.

#### Spatial Heterogeneity and Microenvironmental Factors

The differences in oscillatory patterns at various electrode positions, seen in Figure 2, show the natural diversity in the grape must fermentation system. The 10 mm electrode spacing effectively captures spatial heterogeneity at the scale relevant to fermentation processes: our information-theoretic analysis confirms that each electrode samples a statistically independent microenvironment. Finer spatial sampling would likely reveal additional subcentimeter variations, but our results demonstrate that significant metabolic organization exists even at the centimeter scale. Channel 7 shows the longest average oscillation periods at 2270.65 s, or about 37.8 min. It has the lowest frequency at 0.00044 Hz. This suggests local conditions that support slower metabolic processes or limited substrate availability. The standard deviation of 11366.26 s for Channel 7 shows very irregular oscillations. This might be caused by uneven nutrient transport or changing oxygen levels in that area. Channels 1 and 2 show steady oscillatory patterns. Their period standard deviations are lower, at 152.78 and 266.09 s, respectively. This means they have more stable local microenvironments. The amplitude standard deviation values range from 3.78 to 5.97 mV across channels. This suggests different levels of metabolic intensity. Higher values may link to areas with more microbial activity or active redox conditions.

**Figure 2:**
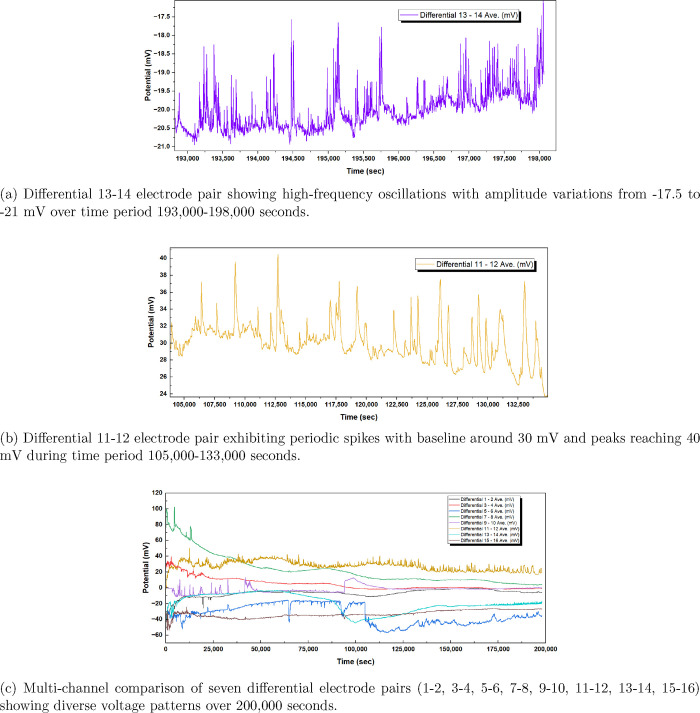
Spontaneous voltage oscillations in grape must (mustalevria) measured using seven differential Pt/Ir electrode pairs (10 mm spacing) at 1 Hz sampling over 200,000 s. (a) Channel 13–14 shows high-frequency fluctuations (3.5 mV peak-to-peak) between 193,000 and 198,000 s. (b) Channel 11–12 exhibits quasi-periodic spike trains (10–12 mV amplitude) during 105,000–133,000 s. (c) Multichannel comparison reveals spatially heterogeneous voltage patterns ranging from −60 to +100 mV across electrode positions, reflecting varied local electrochemical microenvironments during fermentation.

#### Frequency Domain Characteristics and Metabolic Correlations

The characteristic event rates across all channels range from 0.00044 to 0.00215 Hz (mean interevent intervals of 465 to 2271 s). These values represent average time scales of metabolic activity rather than frequencies of periodic oscillations. The actual frequency content of the signals, revealed by power spectral density analysis (Figure 4), shows continuous broad-spectrum power distributed across the measured frequency range (4.348 × 10^–4^ to 2.5 × 10^–3^ Hz) with brown noise characteristics, confirming that dynamics arise from integrated stochastic processes rather than coherent periodic oscillations. These values align with the time scales of cellular metabolic oscillations and are linked to glycolytic pathway dynamics and respiratory cycling in yeast.^44^ The relationship between oscillation dynamics and metabolic state can be conceptually understood through substrate-dependent kinetics. If we hypothesize that oscillation frequency scales with metabolic flux, and flux follows Michaelis–Menten-type kinetics, we can write

$$f_{\mathrm{osc}}(t)\propto \frac{k_{\mathrm{met}}[S](t)}{K_{m}+[S](t)}$$ 3

where *f*_osc_(*t*) represents time-varying oscillation frequency, *k*_met_ is a metabolic rate constant, [*S*]­(*t*) is the time-dependent local substrate concentration, and *K*_m_ is an effective Michaelis constant.^45^ The proportionality symbol (∝) rather than equality emphasizes that this is a simplified conceptual framework—actual oscillation frequency depends on multiple coupled factors beyond substrate concentration alone, including pH, temperature, product inhibition, oxygen availability, and microbial population dynamics.

This framework suggests that spatial variation in oscillation frequencies across channels (ranging from 0.00044 Hz for Channel 7 to 0.00215 Hz for Channel 1, Table 1) could reflect local differences in substrate availability: regions with higher [*S*]­(*t*) would support faster oscillations, while substrate-depleted regions would show slower dynamics. Similarly, the temporal decrease in oscillation frequency as fermentation progresses (visible in Figure 3) could reflect overall substrate depletion as [*S*]­(*t*) decreases over time.

**Figure 3:**
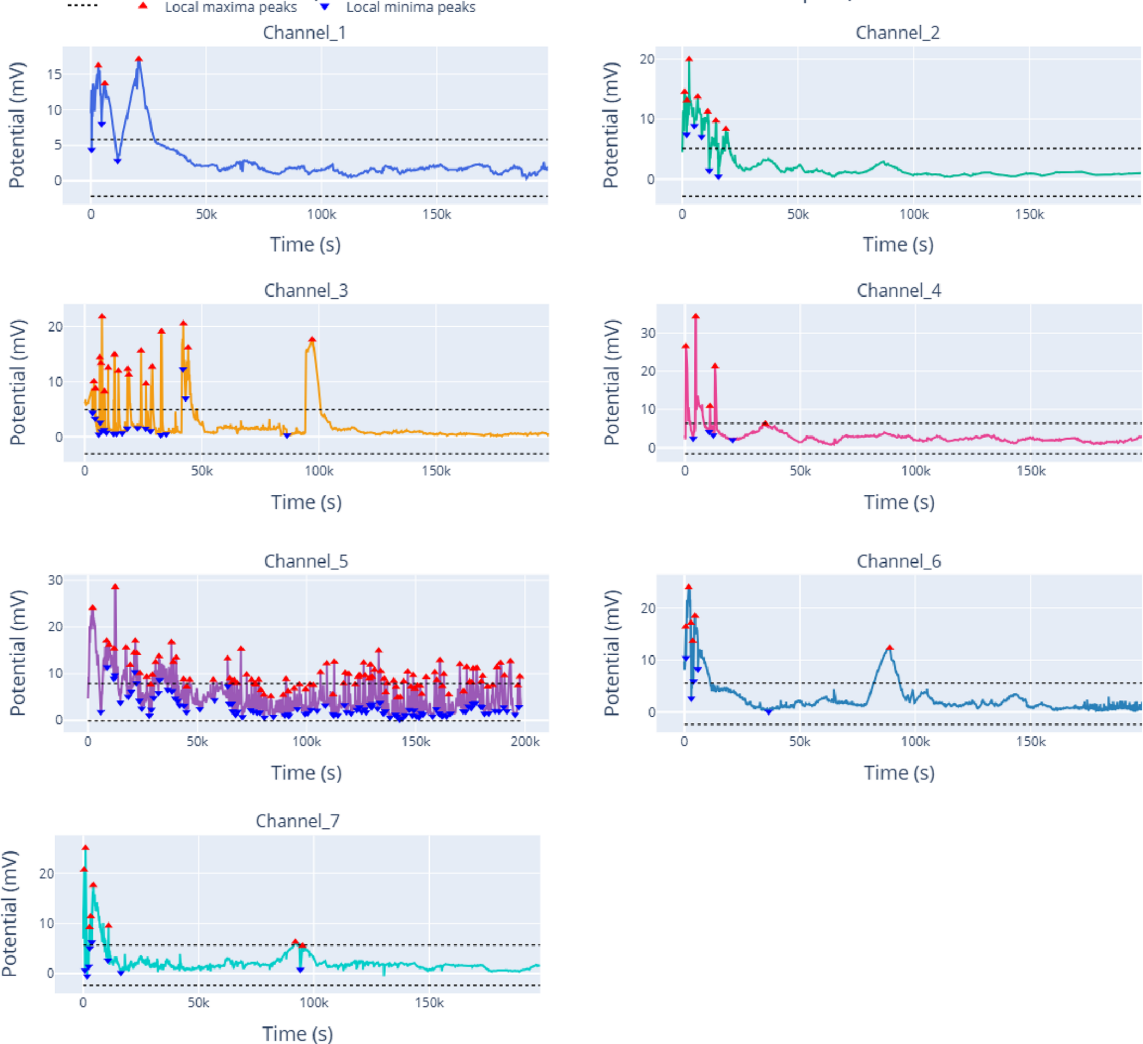
Multichannel spontaneous voltage oscillations recorded during grape-must (mustalevria) fermentation using eight Pt/Ir differential electrode pairs (10 mm spacing) at 1 Hz. Peaks (red markers) were detected automatically using a prominence criterion of 4.0 mV and a minimum spacing of 400 s; black dotted lines indicate the baseline ± prominence band. Distinct spatiotemporal behaviors are observed across channels, including early high-amplitude bursting, intermittent multiphase activity, and sparse long-period oscillations. The transition from fast initial fluctuations to slower oscillatory dynamics reflects evolving metabolic states during fermentation and highlights the spatial heterogeneity of bioelectrochemical activity in the grape must.

However, we emphasize critical limitations of this interpretation: (1) we did not measure [*S*]­(*t*) at any location or time point, so cannot validate this relationship empirically, (2) oscillation frequency may depend on many variables beyond substrate (pH gradients, oxygen levels, metabolite accumulation, microbial community composition), (3) the proportionality constant relating frequency to flux is unknown and may vary spatially and temporally, and (4) different metabolic pathways operate on different time scales, so a single Michaelis–Menten equation cannot capture the full complexity. Without parallel metabolic measurements, this equation serves as a hypothesis-generating framework rather than a validated model.

The range of observed frequencies does suggest the fermentation vessel contains microenvironments operating in different metabolic states: regions supporting faster oscillations likely have better substrate availability, favorable pH, or higher microbial activity, while regions with slower oscillations may experience nutrient limitation or inhibitory conditions. This spatial metabolic heterogeneity is consistent with diffusion-limited transport in the viscous, poorly mixed grape must medium, where convection is minimal and substrate delivery depends primarily on molecular diffusion over centimeter length scales.

The observed frequency range of 0.00044–0.00215 Hz corresponds to periods of 465–2271 s (7.75–37.8 min). These time scales are significantly slower than classical glycolytic oscillations in yeast, which typically exhibit periods of 1–10 min.^45,46^ However, our observations align closely with the time scales of yeast respiratory oscillations (the “ultradian clock”), which typically exhibit periods of approximately 40 min in continuous culture.^44^ Our data also fall within the broader spectrum of cell division cycle-coupled metabolic dynamics, which can range from 40 min up to several hours depending on growth conditions and population synchrony.^47,48^ Given that our system involves heterogeneous natural yeast populations in a spatially structured, diffusion-limited medium rather than homogeneous suspended cultures, these intermediate time scales likely reflect population-level coordination and substrate diffusion limitations rather than pure intracellular glycolytic dynamics. Direct metabolic measurements would be required to definitively distinguish between these mechanisms.

#### Bioelectrochemical Significance and System-Level Implications

The peak detection analysis offers clear data on how bioelectrochemical processes are organized in time and space during grape must fermentation. The average number of peaks per channel is 816/7 ≈ 116.57 peaks. Since each complete oscillation cycle typically includes two peaks (rising and falling excursions from baseline), this represents approximately (116.57 – 1)/2 ≈ 57.8 complete oscillations per channel over 198, 066 s, yielding an average oscillation frequency of 57.8/198, 066 ≈ 2.92 × 10^–4^ Hz per channel, corresponding to a period of approximately 1/(2.92 × 10^–4^) ≈ 3425 s (57 min). This represents a coarse average across all channels and the entire measurement period; individual channels show substantial variation (Table 1), with periods ranging from 762 s (Channel 5) to 2271 s (Channel 7).

Peak detection algorithms help us understand these oscillations. They allow us to compare fermentation systems quantitatively. For example, a decrease in oscillation frequency (longer periods) over time could indicate substrate depletion as fermentable sugars are consumed, with oscillation periods increasing from 500 to 2000 s as glucose/fructose concentrations drop below critical thresholds for maintaining glycolytic oscillations.^45^ Changes in peak amplitude might reflect shifts in yeast population density or metabolic activity intensity—larger amplitudes corresponding to higher cell densities or more vigorous metabolism.^49^ Differences in oscillation patterns between spatial locations (as we observe across our seven channels) could reveal heterogeneous fermentation progression: regions with continued high-frequency oscillations indicate ongoing active fermentation, while regions showing damped or absent oscillations suggest local nutrient exhaustion or inhibitory metabolite accumulation.^50^ Such quantitative oscillation characterization enables objective comparison between different grape varieties, fermentation temperatures, or yeast strains—for instance, comparing whether spontaneous fermentation produces different oscillation signatures than commercial yeast inoculation, or whether temperature-controlled fermentation exhibits more spatially uniform oscillation patterns than ambient-temperature fermentation. This approach transforms qualitative observations (’fermentation seems active’) into quantitative metrics (’oscillation frequency = 0.0015 Hz, amplitude = 8.5 mV’) that can be statistically analyzed and correlated with final fermentation outcomes.

A critical distinction must be drawn between the event-rate metrics derived from peak counting (Table 1) and true oscillation frequencies. Our peak detection does not assume or require periodicity; it simply counts how often voltage exceeds a threshold. The resulting “frequencies” are average rates of these threshold-crossing events. In contrast, genuine periodic oscillations would produce discrete spectral peaks in PSD analysis and regular interevent intervals. Our data show neither: the PSD exhibits continuous power-law spectra (Figure 4), and interevent intervals vary by factors of 5–10 within individual channels (evidenced by large period standard deviations).

**Figure 4:**
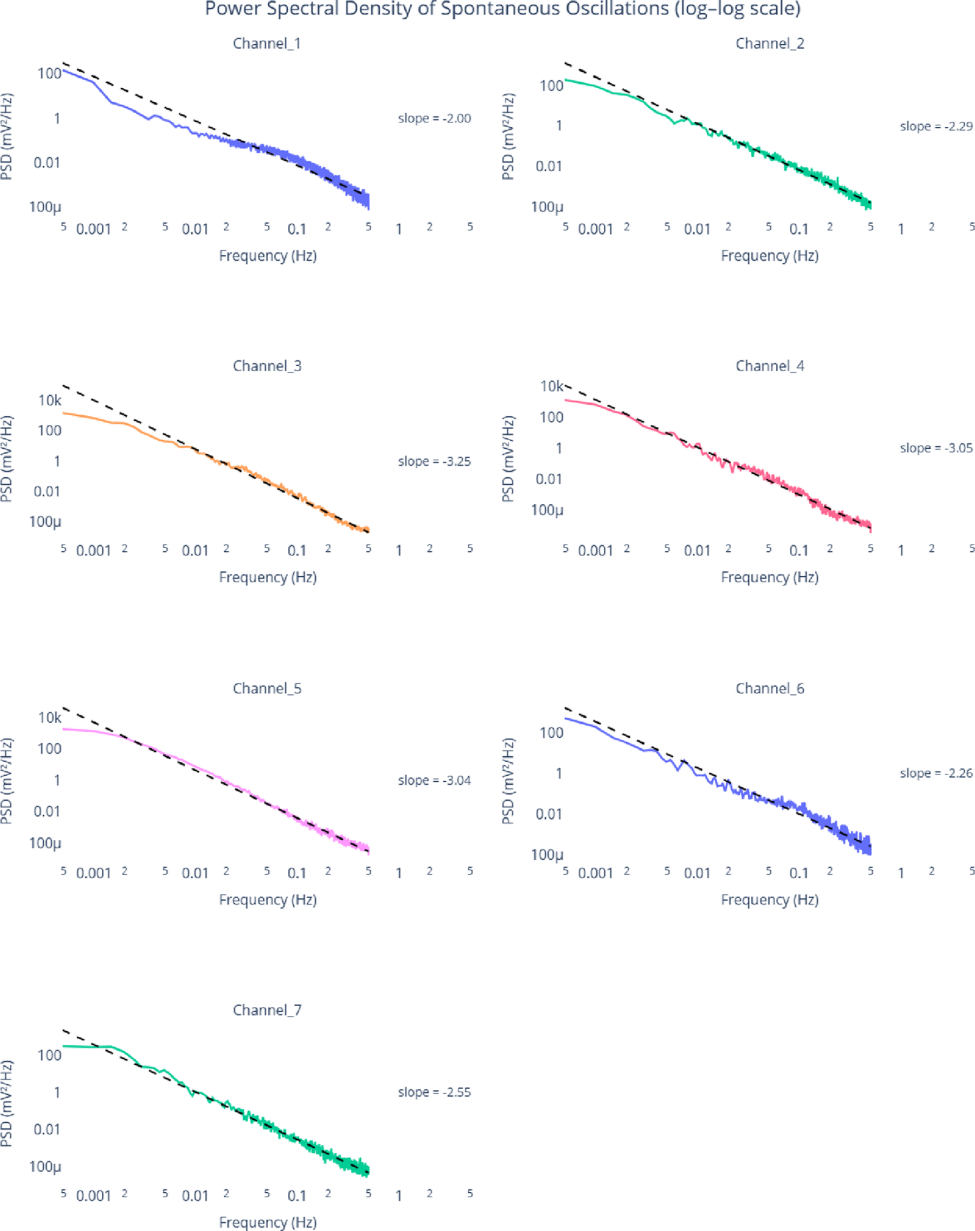
Power spectral density (PSD) analysis of the seven differential electrode channels was performed using Welch’s method, with each subplot showing the log–log frequency structure of spontaneous bioelectrochemical oscillations during grape-must fermentation. Linear fits of the form log­(PSD) = *m*log­(*f*) + *b* reveal distinct spectral regimes across channels. Channels 1–2 exhibit slopes of −2.01 and −2.24, consistent with brown-noise (1/*f* ^2^) behavior and comparatively elevated noise floors (0.00055–0.00032 mV^2^/Hz), indicating strong temporal integration. Channels 3–5 display steeper slopes (−3.04 to −3.28) with much lower noise floors (0.00002–0.00008 mV^2^/Hz), suggesting enhanced low-frequency dominance and more structured metabolic dynamics. Channels 6–7 show intermediate slopes (−2.26 and −2.56), forming a transition between the two regimes. The analysis uses an estimated sampling frequency of 1.00 Hz and highlights the biologically relevant oscillation band spanning 4.348 × 10^–4^ to 2.5 × 10^–3^ Hz.

This irregular behavior is consistent with fermentation operating as a stochastic process driven by random fluctuations in substrate availability, pH, temperature, and microbial activity, rather than as a deterministic oscillator. The biological interpretation is that metabolic “events”—rapid consumption of locally available substrate, coordinated population responses, or redox transients—occur intermittently at characteristic average rates that vary spatially (0.00044–0.00215 Hz across channels), but without the phase coherence or temporal regularity that would constitute true oscillations.

#### Power Spectral Density Characterization of Fermentation Dynamics

The significance of this classification extends beyond mathematical formalism. Environmental changes in ecological systems^30^ often do not follow simple white noise patterns. Instead, natural processes typically exhibit 1/*f*-type spectra with long-range temporal correlations. In our fermentation system (Figure 4), the brown noise characteristics show spectral slopes between −2.01 and −3.28. This suggests that integrative processes mainly drive the dynamics, with metabolic changes building up over time.

This brown noise signature indicates that our system operates far from the white noise regime typically assumed in stochastic models. Brown noise differs fundamentally from white noise: while white noise treats all changes as independent, brown noise exhibits strong memory effects, meaning that past states substantially influence future dynamics. In the context of grape must fermentation, this manifests as cumulative substrate depletion creating long-lasting concentration gradients, progressive metabolite accumulation affecting multiple oscillation cycles, pH drift that gradually shifts the system’s electrochemical baseline, and yeast population dynamics that integrate growth and death over extended periods.

The ecological parallel is instructive. Halley^30^ notes that pink noise (γ = 1) shows the most “democratic” influence across time scales, maintaining equal power per octave from daily to centennial fluctuations. Our system shows a shift toward brown noise (γ > 2), indicating stronger temporal integration. This aligns with closed-system fermentation, where resources are limited and waste products accumulate irreversibly.

This spectral classification has practical implications for process control and optimization. The strong low-frequency components in brown noise imply that short-term measurements may poorly predict long-term behavior. Traditional control strategies based on white noise assumptions may not be sufficient for handling fermentation oscillations. Instead, control algorithms grape must account for the system’s inherent memory and the dominant influence of slow, integrative processes.

Furthermore, the observed power-law scaling in our system, $S(f)\propto \frac{1}{f^{\gamma }}\;\mathrm{with}\;\gamma >2$ , suggests that self-organized criticality may play a role in oscillation dynamics. This phenomenon occurs in complex biological systems when local interactions generate system-wide correlations without external tuning.^51–54^ In fermentation, it likely emerges from the interplay of microbial metabolism, electrochemical gradients, and limited transport processes.

The power spectral density analysis (PSD) in Figure 4 offers key insights into the frequency characteristics of bioelectrochemical oscillations during grape must fermentation. It reveals the noise properties and timing patterns that influence the system’s electrochemical behavior. Using Welch’s method^55^ with appropriate windowing functions provides robust spectral estimates, reduces artifacts from short signals, and minimizes spectral leakage.

The analysis is based on the following formula:

log(PSD)=m·log(f)+b 4

Here, *m* indicates how the power scales with frequency, while *b* represents the overall power level of the oscillations.

This log–log representation is valuable for biological systems because it helps identify power-law scaling relationships found in many natural phenomena, such as neural activity and ecological dynamics. The linear fitting method for the log-transformed data uses least-squares regression to find the best-fit parameters while considering measurement uncertainties and the natural variability in biological signals.

The spectral slope distributions from the seven electrode channels reveal distinct noise patterns that reflect the physical and biochemical processes driving fermentation dynamics. Channels 1 and 2 exhibit spectral slopes of −2.01 and −2.24, values close to the expected brown-noise scaling relationship (1/*f* ^2^), which is typical of systems influenced by random walks or diffusion-limited transport. These channels also show higher noise floors (0.00055 and 0.00032 mV^2^/Hz), suggesting broad-spectrum electrochemical activity. Such activity may originate from diverse metabolic pathways or yeast populations with varying activity levels.

Brown noise indicates strong temporal correlations: past electrochemical states influence current measurements, reflecting memory-like properties in fermentation systems. In these systems, substrate depletion, metabolite accumulation, and pH changes can have lasting effects that persist across several oscillation cycles. The steeper spectral slopes in Channels 3, 4, and 5 (from −3.04 to −3.28) show a unique dynamic. This pattern features stronger low-frequency dominance and greater temporal correlations than classic brown-noise behavior. This spectral signature hints at more organized metabolic processes.

These processes take longer and may connect to how yeast behaves collectively, substrate gradients, or coordinated enzyme actions that occur over time. The lower noise floors in these channels (2 × 10^–5^ to 8 × 10^–5^ mV^2^/Hz) indicate cleaner signals, meaning less high-frequency interference. This might suggest more stable local microenvironments or strong metabolic oscillators that can suppress competing noise sources. The steep slopes suggest that the processes exhibit superdiffusive traits and may be affected by long-range correlations, going beyond simple Markovian dynamics.^56^ This indicates possible collective behavior emerging in specific areas of the fermentation medium. Channels 6 and 7 show intermediate spectral behavior with slopes of −2.26 and −2.56. They connect the two main spectral regimes in the system. This evidence highlights the varied fermentation processes in the grape-must medium. These spectral characteristics suggest that electrode locations sample microenvironments that may exhibit hybrid dynamical properties. The strong correlation between spatial position and spectral slope (ranging from −2.01 to −3.28 across channels separated by only 3–5 cm) indicates that PSD characteristics may serve as fingerprints of local conditions—microbial community structure, substrate availability, or oxygen gradients. This position-dependence limits direct generalizability: different electrode configurations would likely yield different spectral distributions. To extract universal principles, future work should employ replicate fermentations with randomized electrode placement, statistical deconvolution treating position as a random effect, and direct correlation of spectral parameters with microbiological measurements at electrode locations. If spectral signatures reliably indicate local metabolic states, multichannel electrical monitoring could enable real-time spatial mapping of fermentation ecology, informing targeted interventions to optimize process homogeneity and consistency. The noise-floor values for these channels are 6.0 × 10^–4^ and 1.0 × 10^–4^ mV^2^/Hz, showing a difference of nearly 1 order of magnitude. This indicates significant local changes in the balance between clear signals and background noise. This spatial gradient in spectral properties provides clear evidence of the complex three-dimensional structure of fermentation dynamics. Different areas of the medium develop under unique biophysical constraints, yet remain connected through substrate transport and metabolite exchange.

The observed spectral characteristics greatly impact our understanding of information processing in biological fermentation systems. They relate closely to theories of *criticality* and optimal information transfer in complex systems.^57–59^ Brown-noise scaling, with slopes around −2, stands out compared to pink noise, which exhibits a 1/*f* scaling and a slope near −1. This pink-noise behavior is common in systems at the edge of chaos, such as neural networks and adaptive biological systems, which often show optimal information processing.^60^ The steeper slopes observed in some channels suggest that the grape-must fermentation system relies on integrative processes, showing strong temporal correlations and deviating from the balanced dynamics typical of critical behavior. This finding indicates that the fermentation system exhibits complex dynamics and spontaneous oscillations, yet it may not be optimized for rapid information transfer or adaptation to environmental changes. However, this apparent limitation from a conventional computing perspective opens interesting possibilities for unconventional computing and biocomputing research that exploit rather than fight against these natural characteristics.

Computational paradigms suited to fermentation dynamics: Rather than viewing brown noise scaling and slow information transfer as deficits, we can recognize them as inherent computational features. The strong temporal integration (memory effects spanning multiple oscillation cycles) means fermentation naturally performs time-averaging and low-pass filtering—operations that are computationally expensive in digital systems but emerge spontaneously here. The spatial independence between channels combined with global environmental coupling suggests architectures for distributed sensor networks where autonomous nodes respond to shared signals without requiring explicit internode communication. The high-entropy XOR operations indicate natural comparison/difference detection between spatial regions—a primitive operation for pattern recognition tasks.

Research directions for fermentation-based biocomputing:

(1) Controllable state steering: Our state space analysis reveals that the system preferentially occupies certain configurations. Can external perturbations—localized substrate injection, temperature gradients, pH adjustments, or electrical stimulation—controllably steer the system between different attractor states? If so, these states could encode computational outputs, with perturbations serving as inputs to a biological logic system.

(2) Coupled fermentation networks: Could multiple fermentation vessels be coupled (through shared gas headspace, liquid channels, or electrical connections) to create larger computational networks? Our single-vessel system already shows emergent collective behavior; scaling to networked vessels might reveal higher-order computational properties analogous to neural network architectures.

(3) Hybrid biosilicon systems: The continuous electrical signals we measure could interface directly with electronic circuits. Fermentation dynamics could modulate silicon-based processors (bioto-silicon), or electronic stimulation could guide fermentation states (silicon-to-bio), creating hybrid systems combining biological adaptability with electronic precision.

(4) Adaptive and learning behaviors: Does repeated structured perturbation lead to adaptation? If fermentation systems are cyclically exposed to environmental patterns, do their electrical responses change in ways suggesting learning or evolutionary optimization? The brown noise characteristics indicate memory, which is a prerequisite for learning.

(5) Computational complexity characterization: What class of computational problems could fermentation dynamics solve efficiently? The parallel, stochastic nature suggests potential for: optimization problems exploiting natural search of state space, pattern recognition leveraging spatial heterogeneity detection, or time-series prediction using the system’s temporal integration properties. Determining whether fermentation can solve Nondeterministic Polynomial time (NP-complete) problems or perform universal computation (even slowly) would establish its theoretical computing power.

(6) Reservoir computing applications: Fermentation dynamics may be particularly suited to reservoir computing paradigms, where complex nonlinear dynamics serve as a computational substrate without requiring explicit programming. The high-dimensional state space (128 configurations in our seven-channel system), nonlinear dynamics (brown noise), and memory effects (temporal integration) are precisely the properties desired in reservoir computing systems.

The key insight is that biological systems like fermentation need not compete with silicon on speed or efficiency metrics designed for conventional computing. Instead, they may excel at different computational tasks: robust operation in uncontrolled environments, self-repair and adaptation, energy-efficient parallel processing, and integration of sensing with computation. Our characterization of grape must fermentation’s information-processing properties—spatial heterogeneity, temporal correlations, Boolean operations, state space structure—provides the foundational knowledge needed to explore these unconventional computing applications systematically.

#### Environmental Impact on Oscillations

The environmental monitoring data in Figures S2 and 5 show strong links between ambient conditions and bioelectrochemical oscillations in grape must fermentation. This information provides key insights into how external factors influence voltage changes. The time series analysis from July 8–10, 2025, shows significant environmental changes. During this period, the temperature dropped from 34 °C to about 28 °C. Humidity varied between 40 and 50%, following regular daily patterns. The dew point changed between 14 and 20 °C, closely matching humidity shifts.

Environmental changes create a dynamic setting in which the fermentation system grape must adapt to shifting thermodynamic conditions. These fluctuations can influence enzymatic kinetics, microbial metabolism rates, and ionic transport properties, directly affecting the bioelectrochemical signals measured. Documenting these parameters enables us to evaluate how the environment impacts fermentation beyond simple observations, helping to establish clear connections between external conditions and system behavior.

The correlation analysis shows that temperature is the main environmental factor affecting oscillatory behavior in all seven electrode channels. The positive correlation coefficients range from 0.245 in Channel 3 to 0.558 in Channel 2. This indicates that higher temperatures boost bioelectrochemical activity in the fermentation medium. Elevated temperatures accelerate enzymatic reactions and increase molecular movement, which also enhances ion mobility. Together, these effects promote more intense metabolic processes and stronger electrical signals.

The strongest temperature correlations were found in Channels 1, 2, and 6, with values of 0.547, 0.558, and 0.585, respectively. This suggests that these electrode positions are located in areas sensitive to temperature changes, possibly due to high yeast cell density or active metabolic zones where temperature affects the local electrochemical environment.

The moderate correlation observed in Channel 3 (0.245) shows that temperature sensitivity varies across the system. Local factors such as substrate availability, pH buffering capacity, or physical barriers may modulate how bioelectrochemical processes respond to temperature changes.

Humidity has a negative effect on oscillatory activity in all channels. The correlation coefficients range from −0.052 to −0.245. This indicates that higher humidity levels tend to reduce bioelectrochemical oscillations in the grape must system. This surprising finding may reflect how atmospheric moisture, evaporation rates, and dissolved species interact within the fermentation medium. Higher humidity lowers water loss from the grape must surface, potentially diluting solute concentrations and weakening the forces driving electrochemical reactions. Elevated humidity can also alter the partial pressure of fermentation products such as ethanol and carbon dioxide, shifting the balance of important metabolic pathways and affecting the medium’s electrical properties. Channel 2 shows the strongest negative humidity correlation at −0.245. This suggests that this electrode position may be more influenced by surface phenomena or located in a region where concentration effects strongly impact local electrochemical activity.

Dew point correlations show positive values between 0.191 and 0.491. This indicates that dew point integrates the effects of temperature and humidity and reflects the moisture content of the air. The moderate positive correlations suggest that higher dew points correspond to greater absolute humidity at the same temperature. This may enhance certain fermentation activities and highlight the thermal component inherent in the dew point calculation. Channel 1 shows the highest dew point correlation at 0.491, followed by Channel 6 with 0.423. This suggests that these electrode positions respond strongly to changes in environmental moisture. The effects of dew point involve several factors: it influences water activity in the fermentation medium, alters the gas–liquid balance of volatile compounds, and can modify surface tension and wetting properties. These factors are critical for the interfaces between the electrode and medium, as well as for ionic transport near the electrode surfaces.

Environmental correlation patterns show how bioelectrochemical fermentation systems respond to their surroundings. Spontaneous oscillations are not random. They arise from complex interactions between biological processes and external conditions. Positive temperature correlations across all channels show that the grape must system is sensitive to temperature. This means that heat boosts metabolic activity and electrical signals. So, there could be opportunities for using temperature control to optimize fermentation processes.

Temperature control is already standard practice in industrial fermentation, with precise regulation typically maintained within ±0.5 °C for consistency. Our contribution is not suggesting temperature control per se, but rather demonstrating that continuous electrical monitoring can detect temperature effects on fermentation dynamics in real-time without chemical sampling. The quantified correlation strengths (*r* = 0.245–0.585) could enable temperature-correction algorithms for electrical monitoring systems: rather than treating voltage changes as solely reflecting fermentation progress, monitoring systems could computationally adjust for ambient temperature fluctuations to extract the underlying metabolic signal. This would be particularly valuable for traditional or small-scale fermentations where precise temperature control is impractical or economically infeasible, but where understanding temperature’s influence on electrical signatures could improve process interpretation. Additionally, the spatial heterogeneity in temperature sensitivity across channels (correlation coefficients vary by more than 2-fold) suggests that multichannel electrical monitoring could detect localized temperature gradients or cooling inefficiencies in large fermentation vessels—information not available from single-point temperature probes.

Inverse humidity relationships show how evaporation and concentration affect electrochemical conditions. Intermediate dew point correlations show that absolute moisture content affects system behavior. Temperature and relative humidity also play a role. These findings demonstrate that bioelectrochemical oscillations can serve as environmental-coupling indicators in fermentation monitoring. While temperature effects on fermentation kinetics are well-established, the specific correlation strengths we quantify (*r* = 0.245–0.585 for temperature, negative correlations for humidity) provide calibration data for interpreting electrical measurements under varying ambient conditions. Unlike traditional end point measurements (final ethanol concentration, cell counts), continuous voltage monitoring reveals how environmental fluctuations modulate metabolic dynamics in real-time. This suggests electrical signals could enable adaptive process control that compensates for environmental variations—for instance, adjusting aeration or cooling based on temperature-corrected oscillation frequency rather than waiting for chemical analysis. The novelty lies not in discovering environmental effects, but in establishing that electrical oscillations encode this information continuously and noninvasively, offering a complementary monitoring modality to conventional chemical and microbiological methods. This is especially important for applications requiring consistent signal characteristics across different environments, such as industrial fermentation monitoring in facilities with seasonal temperature variations, quality control systems that must operate reliably across different production batches and geographic locations, or bioelectrochemical sensors deployed in field settings where ambient conditions cannot be controlled. For instance, a fermentation monitoring system calibrated at 25 °C might produce systematically different voltage signatures at 30 °C due to the strong temperature correlations we observe (*r* = 0.245–0.585), potentially leading to misclassification of fermentation state if environmental effects are not accounted for in the signal processing algorithms.

As shown in Figure 5, all temperature correlations display three asterisks (***), indicating *p* < 0.001, and demonstrating extremely high statistical significance for the relationship between temperature and bioelectrochemical oscillations across all seven channels (correlation coefficients ranging from 0.180 to 0.915). In contrast, dew point and humidity correlations across all channels show no asterisks or very high *p*-values (*p* > 0.05), indicating that these relationships are not statistically significant, despite some channels exhibiting nonzero correlation coefficients (e.g., humidity *p* ≈ 0.13–0.84 for all channels).

**Figure 5:**
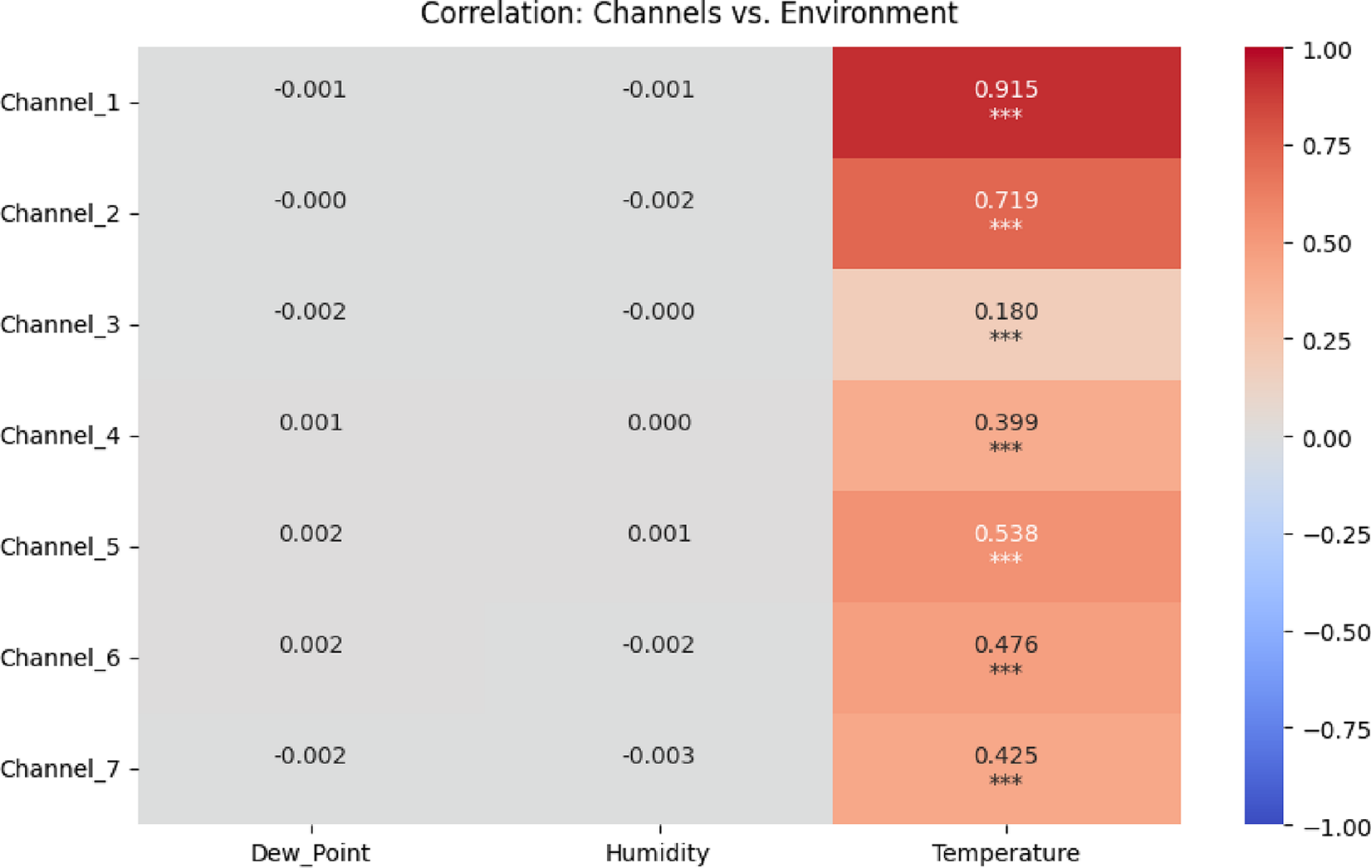
Correlation heatmap showing Pearson correlation coefficients between spontaneous voltage oscillations in seven electrode channels and environmental factors (dew point, humidity, and temperature). Statistical significance: *** indicates *p* < 0.001. Temperature exhibits the only statistically significant correlations (*r* = 0.180–0.915, all *p* < 0.001), while dew point and humidity correlations are nonsignificant (*p* > 0.05 for all channels). The fermentation system’s oscillatory behavior responds strongly to temperature variations but remains robust to changes in ambient humidity and dew point.

#### Boolean Logic Gate Implementation in Bioelectrochemical Oscillations

The Boolean operations described here are analytical tools applied computationally to recorded voltage data, not physical hardware implementations. After acquiring continuous voltage signals via the ADC-24, we binarize the data using threshold criteria and then apply Boolean logic operators in software to characterize timing relationships between spatially separated electrode pairs.

Turning continuous voltage waves into discrete Boolean states allows us to analyze grape must fermentation. This approach uses digital logic operations, creating a new way to understand how biological electrochemical systems process information. The binarization process follows a threshold-based criterion, where each channel’s voltage signal *V*_*i*_(*t*) is converted to a binary state *B*_*i*_(*t*) according to

$$B_{i}(t)=\{\begin{array}{ll} 1 & \mathrm{if}\;V_{i}(t)>V_{\mathrm{threshold}},\\ 0 & \mathrm{if}\;V_{i}(t)\leq V_{\mathrm{threshold}} \end{array}$$ 5

where *V*_threshold_ = 2.0 mV represents the discrimination level separating high (active) and low (inactive) electrochemical states. This binary representation reduces the complexity of continuous oscillatory signals while preserving the essential temporal patterns of metabolic activity, analogous to neural spike train analysis where action potentials are represented as discrete events.

Using six basic Boolean logic gates (AND, OR, XOR, NAND, NOR, XNOR) on all unique channel pairs helps us understand interchannel relationships. This approach reveals the computational properties that arise from the fermentation system’s changes over time and space.

Boolean gate operations are defined for two binarized channels, *B*_A_(*t*) and *B*_B_(*t*), using standard logical operators. Entropy and transition-rate metrics measure the output complexity. The gate outputs are calculated as^61,62^

$$\begin{array}{ll} \mathrm{AND}(t) & =B_{A}(t)\wedge B_{B}(t),\\ \mathrm{OR}(t) & =B_{A}(t)\vee B_{B}(t),\\ \mathrm{XOR}(t) & =B_{A}(t)⊕B_{B}(t),\\ \mathrm{NAND}(t) & =\neg (B_{A}(t)\wedge B_{B}(t)),\\ \mathrm{NOR}(t) & =\neg (B_{A}(t)\vee B_{B}(t)),\\ \mathrm{XNOR}(t) & =\neg (B_{A}(t)⊕B_{B}(t)) \end{array}$$ 6

The information content of each gate output is quantified using Shannon entropy:

$$H=-\underset{i}{\sum }p_{i}\log_{2}\left(p_{i}\right)$$ 7

where *p*_*i*_ represents the probability of state *i* ∈ {0, 1}. Entropy values range from 0 bits (completely predictable) to 1 bit (maximally random). The transition rate measures how often the gate output changes state; it helps to understand the timing of logical operations and whether channel pairs are synchronized or desynchronized.

Analysis of all 21 channel pairs shows that the mustalevria system exhibits diverse logical relationships. Average gate-specific statistics reveal that different spatial electrode configurations create unique Boolean functions through bioelectrochemical coupling. This suggests new opportunities for unconventional computing based on biological fermentation substrates.

The binary transformation of voltage oscillations shows different activation patterns in the seven electrode channels, as seen in Figure S3. Channel 5 kept switching rapidly during the whole measurement, recording 260 peaks—the highest among all channels (Table 1)—with sustained activity throughout the full 200,000 s period. In contrast, Channel 2 collapsed early, going quiet after about 100,000 s, accumulating only 38 peaks total. Multiple lines of evidence indicate these patterns reflect biological heterogeneity rather than electrode malfunction: both channels exhibited normal behavior in pre-experiment buffer tests, Channel 2 showed typical oscillatory activity before declining (indicating initial functionality), Channel 5 maintained consistent temperature correlation throughout (*r* = 0.343, Figure 7), and postexperiment impedance measurements confirmed electrode integrity. The contrasting behaviors are consistent with spatial gradients in substrate availability—Channel 2’s peripheral position likely experienced earlier nutrient depletion than Channel 5's more central location. Nevertheless, parallel metabolic measurements would be needed to definitively confirm this interpretation. This contrast shows that Channel 5 samples a stable microenvironment with steady substrate availability. In contrast, Channel 2 likely tracks an area where substrate runs low or inhibitory metabolites build up. The binary transformation of voltage oscillations shows different activation patterns in the seven electrode channels, as seen in Figure 8. Channel 5 kept switching rapidly during the whole measurement, recording 260 peaks—the highest among all channels (Table 1)—with sustained activity throughout the full 200,000 s period. In contrast, Channel 2 collapsed early, going quiet after about 100,000 s, accumulating only 38 peaks total.

The 2.0 mV threshold separates signal from noise. Yet, this binary method loses amplitude details. For example, a 5 mV spike and a 15 mV spike both turn into the same “1” state in this system. This limitation has several consequences for our analysis: (1) we cannot distinguish between weak and strong metabolic events, only their presence or absence, (2) gradual changes in metabolic intensity appear as abrupt on/off transitions, potentially missing important intermediate states, (3) Boolean gate statistics reflect only timing relationships (when channels are active) rather than coupling strength (how strongly they covary in amplitude), and (4) entropy calculations depend entirely on state occupancy probabilities rather than the magnitude of signals. However, this limitation primarily affects quantitative interpretation rather than qualitative conclusions—the spatial independence we observe (low mutual information) and temporal asynchrony (high XOR entropy) would persist regardless of amplitude information, since they fundamentally reflect timing rather than magnitude relationships. Alternative approaches preserving amplitude information, such as continuous mutual information or correlation analysis, provide complementary perspectives and largely corroborate the Boolean analysis findings.

Figure S4 shows how the six basic logic gates pull unique features from the time relationship between Channels 1 and 2. The AND gate has a low activation percentage of 18.47% and low entropy at 0.69 bits. This shows that high-state conditions are rare. Electrodes far apart activate together only during major metabolic events. They can also connect if their environments share substrate transport or electrical fields. The XOR gate has a high entropy of 0.853 bits and an activation percentage of 27.85%. This effectively measures temporal asynchrony, which is when one channel is active while the other is quiet. The transition rate metric needs careful review. Values near 0.002 show state changes happen about once every 500 s. This is quite slow compared to the oscillation frequencies found in our peak detection analysis, which range from 465 to 2271 s. This suggests that binarization might smooth out quick changes in amplitude. As a result, it could miss key dynamic information that stays above or below the threshold. This mismatch between transition rates (0.002 Hz, one change per 500 s) and oscillation periods (465–2271 s from peak detection) reveals that Boolean binarization captures a subset of fermentation dynamics—specifically, threshold-crossing events—rather than the full oscillatory behavior. The implication for fermentation process analysis is that binary state monitoring would detect major metabolic transitions (shifts between active and quiescent phases) but miss continuous fluctuations within each phase. For instance, gradual substrate depletion might cause oscillation amplitude to slowly decrease while remaining above threshold, appearing as sustained “1” state in Boolean analysis despite changing metabolic intensity. This means Boolean methods are most suitable for detecting discrete fermentation stages (lag phase ending, exponential growth beginning, stationary phase) rather than tracking continuous metabolic flux rates. For real-time process control applications, hybrid monitoring combining binary state detection (for stage identification) with amplitude analysis (for intensity quantification) would provide more complete information than either approach alone. The Boolean framework’s value lies in revealing spatial coordination patterns and system-level organization rather than providing detailed metabolic kinetics.

Table 2 shows data from 21 channel pairs. It highlights clear patterns in how logic gates behave. The XOR/XNOR pair shows the highest entropy at 0.93223 bits and top transition rates of 0.00299. These gates capture the different timing and asynchronous dynamics in fermentation processes that are spread out in space. This finding matches what we expect: XOR operations respond to phase differences and timing mismatches. These are key traits when tracking independent metabolic oscillators that work at different frequencies or phases.^47,63–65^ The AND/NAND gates have low entropy at 0.71434 bits. This means that activating random electrode pairs happens less often and is more predictable. The symmetry in complementary gate pairs (AND/NAND, OR/NOR, XOR/XNOR) is crucial. They have the same transition rates and entropies, showing the same timing but with opposite polarity. Yet, this mathematical symmetry means that complementary gate pairs provide redundant information about the system—analyzing NAND adds nothing beyond what AND already reveals, since they have identical transition rates and entropies differing only in output polarity. Consequently, the six Boolean gates collapse to three independent characterizations of interchannel relationships: AND/NAND quantifies coactivation probability, OR/NOR measures the probability that at least one channel is active, and XOR/XNOR captures temporal asynchrony. We retain all six in our analysis for completeness and consistency with standard Boolean logic frameworks, but recognize that the fundamental information content comes from three distinct coupling modes rather than six independent operations.

**Table 2:** Average Boolean Logic Gate Statistics Calculated across All 21 Unique Channel Pairs (Combinations of 7 Channels Taken 2 at a Time) in the Grape Must Fermentation System^a^

**gate**	**activation percentage (%)**	**transition rate**	**entropy (bits)**
AND	22.39524	0.00122	0.71434
OR	65.65905	0.00178	0.79957
XOR	43.26333	0.00299	0.93223
NAND	77.60476	0.00122	0.71434
NOR	34.34095	0.00178	0.79957
XNOR	56.73667	0.00299	0.93223

a Each metric represents the mean value from gate operations on all pairwise combinations of differential electrodes during the 200,000-second measurement period. The activation percentage indicates how often each gate output remained active (1), reflecting the common logical conditions present in the spatial layout of the electrodes. The transition rate measures how often state changes occur per sample; higher values indicate more dynamic relationships between channel pairs. Shannon entropy quantifies the information content of the gate outputs: values close to 1 bit indicate balanced probabilities between binary states (high unpredictability), whereas lower values indicate biased outputs and reduced information capacity. Complementary gate pairs—such as AND/NAND, OR/NOR, and XOR/XNOR—exhibit identical transition rates and entropies because of their symmetric logical definitions, differing only in output polarity. The XOR and XNOR gates show the highest average entropy at 0.93223 bits and transition rates of 0.00299. This suggests that these operations best capture timing differences and dynamic interactions in fermentation microenvironments that are spatially separated. Conversely, AND and NAND gates display the lowest transition rates (0.00122) and entropies (0.71434 bits). This indicates that high-state activation across channel pairs is rare and predictable within the fermentation system, aligning with the heterogeneous metabolic activity observed in the grape must medium.

The correlation heatmap in Figure 6 shows interchannel synchronization. Most values are below 0.45, which means there are mostly weak to moderate correlations. This suggests significant spatial independence in the fermentation system. Channels 6 and 7 have a strong correlation of 0.44. This likely means they are close together, share metabolic links, or have similar local environments. The Pearson correlation coefficient has big limits when used with binary data. It treats the 0/1 states like continuous variables. This can lead to misleading results, especially if the distributions are skewed or if there are phase shifts between signals. The near-zero correlation between Channels 5 and 6 (−0.033) is intriguing. Channel 5's steady activity does not seem linked to Channel 6’s bimodal distribution. This suggests that these electrode positions measure different metabolic processes with little interaction.

**Figure 6:**
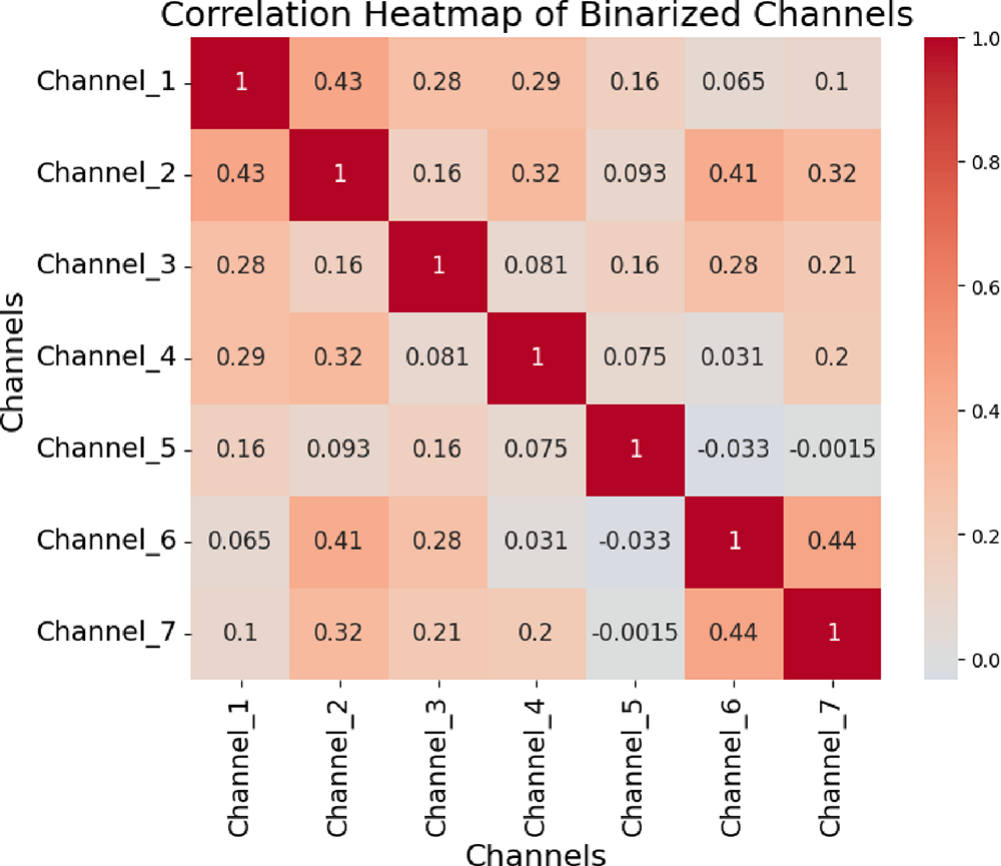
Pearson correlation matrix for binarized states across all seven electrode channels. The correlation coefficients show how well binary activation patterns synchronize over time. They range from −0.033, indicating a slight anticorrelation between Channels 5 and 6, to 0.44, which shows a moderate positive correlation between Channels 6 and 7. Strong diagonal elements (correlation = 1.0) represent self-correlation. The varied correlation structure reveals a complex layout of bioelectrochemical activity. Channels 1 and 2 have a moderate correlation of 0.43, suggesting some synchronized metabolic activity in their regions. Channel 5, however, shows weak or nearly zero correlations with most others, indicating that it behaves more independently. Channels 6 and 7 display the highest correlation at 0.44, which may indicate spatial proximity or similar metabolic influences. Overall, the weak-to-moderate correlations (most below 0.45) suggest that the fermentation system operates with substantial spatial independence. Local electrochemical processes at different electrode sites develop according to their own microenvironments rather than in complete synchrony with the entire system.

The information-theoretic properties revealed by Boolean analysis have direct implications for understanding fermentation organization. High XOR entropy (0.93 bits) indicates that spatially separated regions activate asynchronously—metabolically, this suggests independent timing of substrate consumption bursts, pH oscillations, or redox cycles at different locations rather than synchronized vessel-wide dynamics. The low mutual information between channels (<0.206 bits) quantifies spatial independence: knowing the metabolic state at one location provides minimal predictive information about distant locations, confirming that the fermentation operates as a collection of loosely coupled microenvironments rather than a homogeneous system. The nonuniform state space occupancy (Figure 11) reveals that certain multichannel activation patterns occur preferentially—biologically, this could reflect: (1) spatial organization imposed by substrate diffusion gradients, where certain geometric patterns of active/inactive regions are thermodynamically favorable, (2) metabolic synchronization through diffusible signals (CO_2_, ethanol, organic acids) that couple nearby but not distant regions, or (3) constraints from vessel geometry and convection patterns that create reproducible spatial metabolic structures. The moderate AND gate entropy (0.71 bits) indicates that simultaneous activation of channel pairs is relatively rare and predictable—suggesting that regions rarely experience coordinated metabolic bursts, consistent with substrate competition where active metabolism at one location depletes local resources and inhibits nearby activity. These information-theoretic metrics transform qualitative observations about fermentation heterogeneity into quantitative measures of spatial organization, coordination, and independence that can be compared across fermentation conditions, substrates, or vessel designs.

#### System-Level Computational Analysis through State Space and Logic Gate Entropy

The system-wide binary configurations shown in Figure 7 highlight key principles that govern how the seven-channel fermentation system works together. Instead of exploring all possible system states uniformly (with 7 binary channels, there are 2^7^ = 128 possible configurations), the system exhibits preferential occupancy of specific collective modes. For example, the configuration “1101000” (Channels 1, 2, and 4 active, others inactive) represents one specific state out of the 128 possibilities. The main state appears about nine times more often than expected if activation were random. This nonuniform state distribution can be quantified through the occupancy probability *P*(*s*) for each state *s*, where the Shannon entropy of the state distribution is defined as

$$H_{\mathrm{state}}=-\overset{128}{\underset{s=1}{\sum }}P\left(s\right)\log_{2}\;P\left(s\right)$$ 8

**Figure 7:**
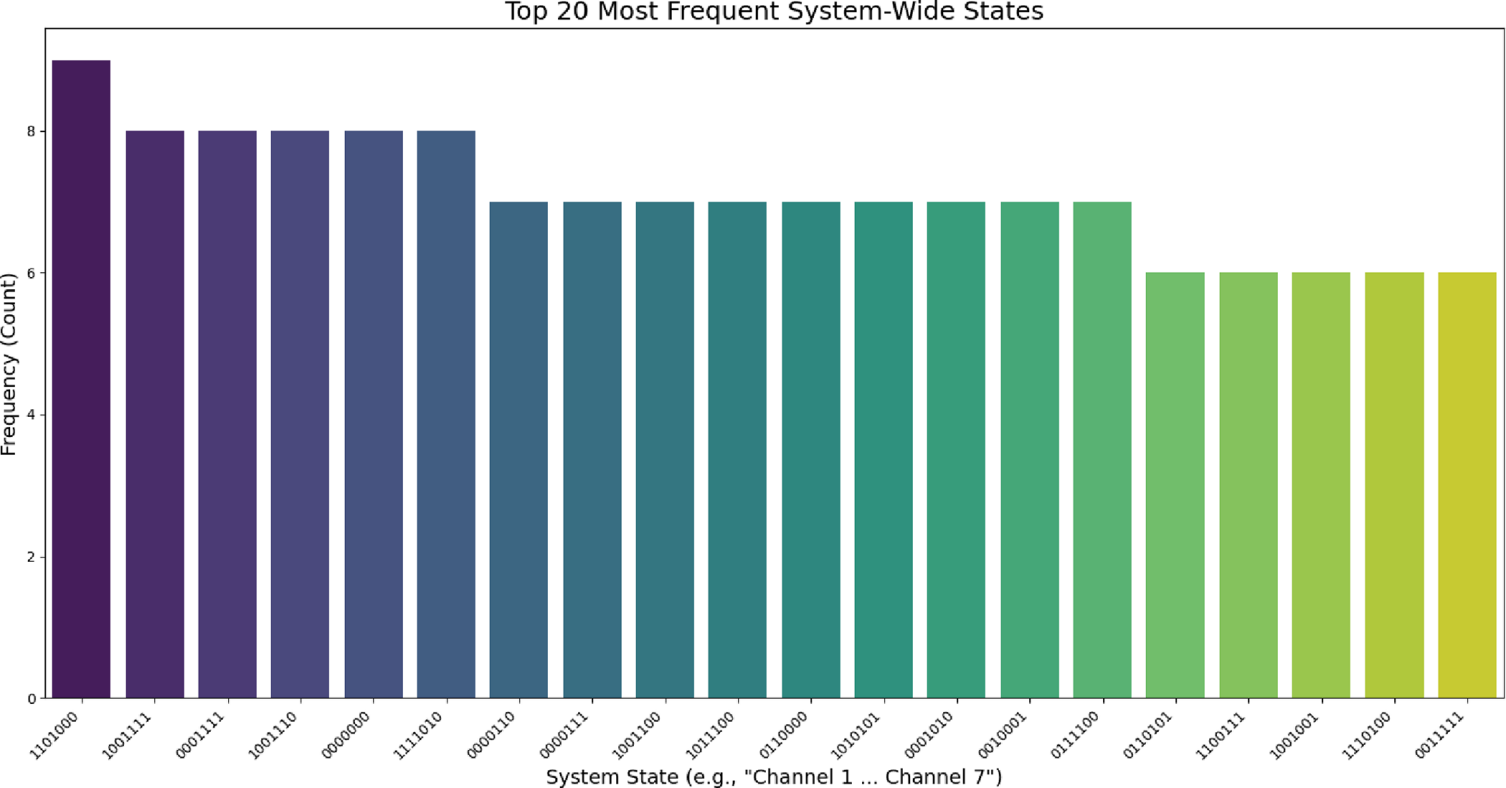
Distribution of the 20 most frequent seven-channel binary configurations during grape must fermentation (total 128 possible states). Each bar represents a unique activation pattern (e.g., “1101000” = Channels 1, 2, 4 active; others inactive). Nonuniform state occupancy indicates preferential collective modes rather than random exploration of configuration space, with the dominant state occurring 9 times per 1000 samples. Sparse activation patterns (few 1 s) are more common than high-activation states, consistent with metabolic resource constraints.

This entropy provides a measure of the system’s effective dimensionality. For a uniform distribution across all states, *H*_state_ = log_2_(128) = 7 bits. However, the observed concentration of probability in certain configurations shows much lower entropy, meaning that fermentation dynamics operate within a limited range of possible configurations. This distribution hints at attractor basins in the system’s phase space: certain activation patterns form stable or metastable states that arise from metabolic constraints. High-activation states, where many channels are in state “1,” are rare compared to sparse states. This scarcity likely relates to energy requirements: metabolic bursts need abundant substrates and coordinated activity among yeast populations in different regions. Such conditions occur less frequently than quieter or more localized states.

The box plot in Figure 8 shows gate-specific entropy values. This helps us understand the pairwise computational properties that shape the overall behaviors observed in the state space analysis. The XOR and XNOR gates show similarly high entropy for all 21 channel pairs. The median value for *H*_XOR_ is about 0.99 bits, and the interquartile ranges are less than 0.05 bits. This uniformity shows that temporal asynchrony—where one channel is active while another is not—is a universal trait of the fermentation system, regardless of spatial separation or local environmental differences.

**Figure 8:**
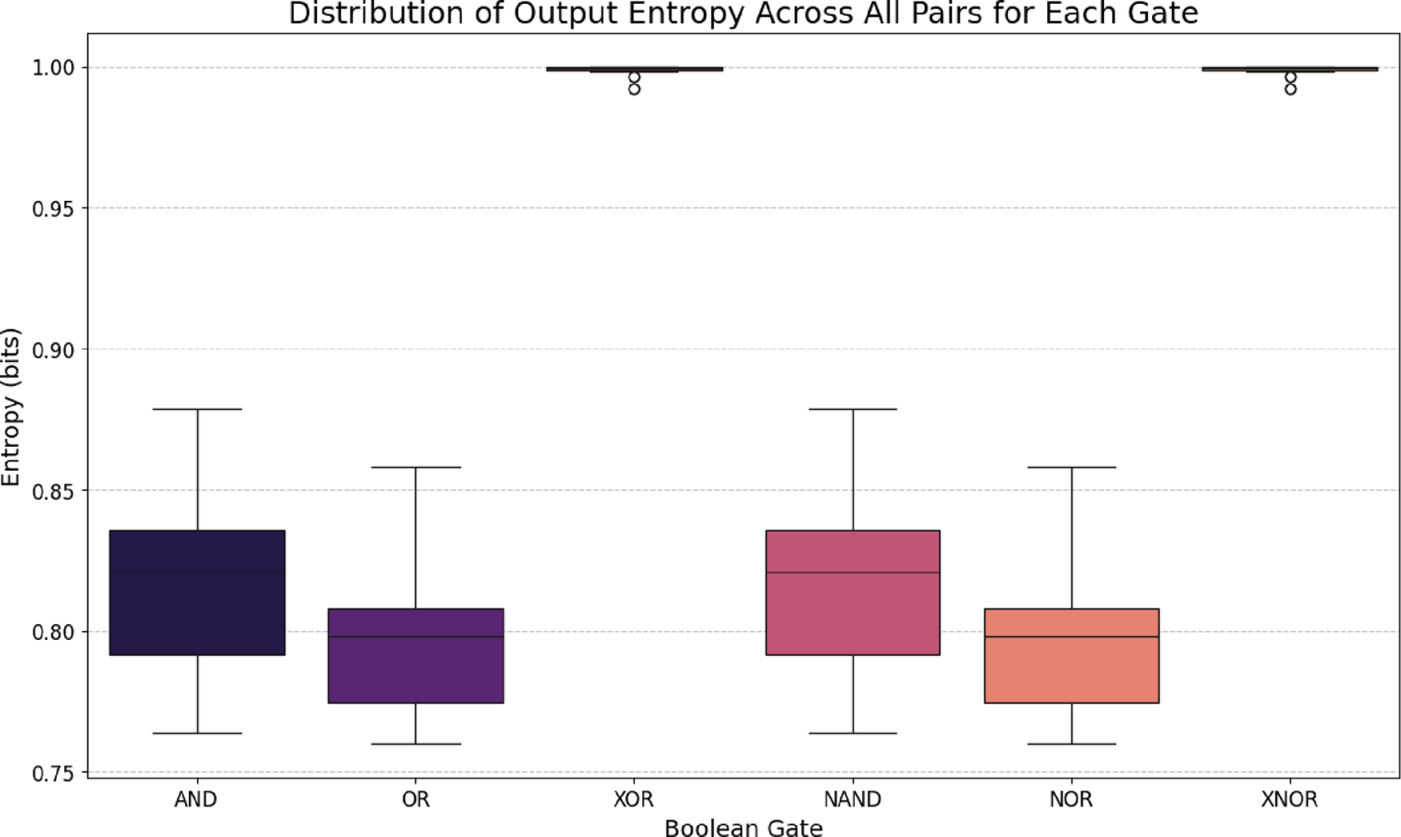
Box plots show the Shannon entropy of six Boolean logic gates computed from all 21 electrode channel pairs. Entropy ranges from 0 (predictable) to 1 (random), with boxes marking the 25th–75th percentiles and whiskers showing the full nonoutlier range. XOR and XNOR gates exhibit consistently high entropy (∼0.98–1.0 bits) with very low variance, indicating that channel pairs generate highly balanced, largely independent outputs across the system. AND and NAND gates show lower median entropy (∼0.80–0.83 bits) and much greater variability, reflecting differences in how often specific channel pairs coactivate. OR and NOR gates fall in an intermediate range but display distinct spread patterns. Overall, the persistent high entropy of XOR/XNOR confirms that temporal activity across electrodes is largely asynchronous and unsynchronized. These results suggest that the fermentation medium behaves as a distributed computational substrate capable of both predictable (e.g., AND-like) and stochastic (e.g., XOR-like) information processing depending on the logical operation applied.

The mathematical expectation for XOR gate entropy can be derived from the joint probability distribution of two binary channels:

$$H_{\mathrm{XOR}}=-p_{10}\log_{2}\left(p_{10}\right)-p_{01}\log_{2}\left(p_{01}\right)-p_{00}\log_{2}\left(p_{00}\right)-p_{11}\log_{2}\left(p_{11}\right)$$ 9

where *p*_*ij*_ represents the probability of finding channel A in state *i* and channel B in state *j*. Maximal entropy (*H* = 1 bit) occurs when the XOR output distribution is perfectly balanced, requiring

$$p_{10}+p_{01}=p_{00}+p_{11}=0.5$$ 10

The near-maximal entropy observed in all channel pairs is notable: even though there are different levels of pairwise correlation (see Figure 6), the timing of state changes remains statistically independent. This independence prevents the system from synchronizing, which would otherwise reduce XOR entropy.

The behavior of AND and NAND gates is different. They show a median entropy between 0.80 and 0.83 bits, but with a larger variance across channel pairs. This variation highlights differences in coactivation probabilities within the fermentation medium. For the AND gate, entropy peaks when the probability of both inputs being active, *p*_11_, balances with the total probability of the other states, *p*_00_ + *p*_01_ + *p*_10_. The wide range of AND gate entropy values, from about 0.76 to 0.88 bits, shows that some electrode pairs rarely activate together, leading to low-entropy outputs, whereas others show more balanced activation patterns.

This spatial heterogeneity can be quantified using the coefficient of variation:

$${\mathrm{CV}}_{\mathrm{gate}}=\frac{\sigma_{H}}{\mu_{H}}$$ 11

where σ_H_ and μ_H_ represent the standard deviation and mean entropy across all pairs for a given gate. The AND/NAND gates have an average coactivation probability of about 0.04, which is much higher than that of the XOR gates (around 0.01). This demonstrates that coactivation probabilities vary greatly across spatial locations. However, asynchronous switching remains consistent everywhere. These findings suggest that the fermentation system exhibits local independence in its temporal patterns, as reflected by the consistent XOR entropy, while also showing structured spatial patterns of metabolic coordination, indicated by the variability of AND gate behavior. Such spatial structure likely arises from factors such as substrate diffusion gradients, pH zoning, or organized yeast colony distributions.

The analyses reveal two key traits: uniform asynchrony (XOR) and diverse coactivation (AND). This suggests that the grape must fermentation system functions as a distributed processor, operating in different ways simultaneously. The state space analysis shows that this computational substrate does not explore its configuration space evenly; instead, it exhibits structured collective dynamics with certain preferred attractor states. We note that this behavior is specific to our grape must fermentation system with its particular microbial composition, substrate concentrations, and preparation method; different fermentation substrates, microbial strains, or environmental conditions may exhibit different state space structures and occupancy patterns. The gate entropy distributions reveal key logical operations underlying these behaviors. XOR operations capture maximal information about timing differences between channel pairs, leading to high entropy and unpredictability. In contrast, AND operations encode specific spatial information about metabolic coordination, with predictability varying according to the electrode configuration. This duality can be formalized using the *mutual information* between channels *A* and *B* (Figure 9):

I(A;B)=H(A)+H(B)−H(A,B) 12

where *H*(*A*) and *H*(*B*) denote the Shannon entropies of the individual channels, and *H*(*A*,*B*) is their joint entropy. High XOR entropy combined with low mutual information (mean *I*(*A*;*B*)*I*(*A*;*B*)*I*(*A*;*B*) = 0.012 bits; Table 3) indicates statistical independence between channels, while variations in AND entropy signal location-dependent metabolic coupling. These analyses show that traditional food fermentation systems behave as complex adaptive systems. Local processing elements—such as electrode pairs performing Boolean operations—give rise to global patterns like system-wide state distributions. This occurs through distributed, partly independent dynamics rather than centralized control. Such findings extend beyond fermentation science: they suggest that biological electrochemical systems may employ unique computational mechanisms, where information is not stored in single-channel states but instead resides in the statistical patterns of group configurations and their logical relationships.

**Figure 9:**
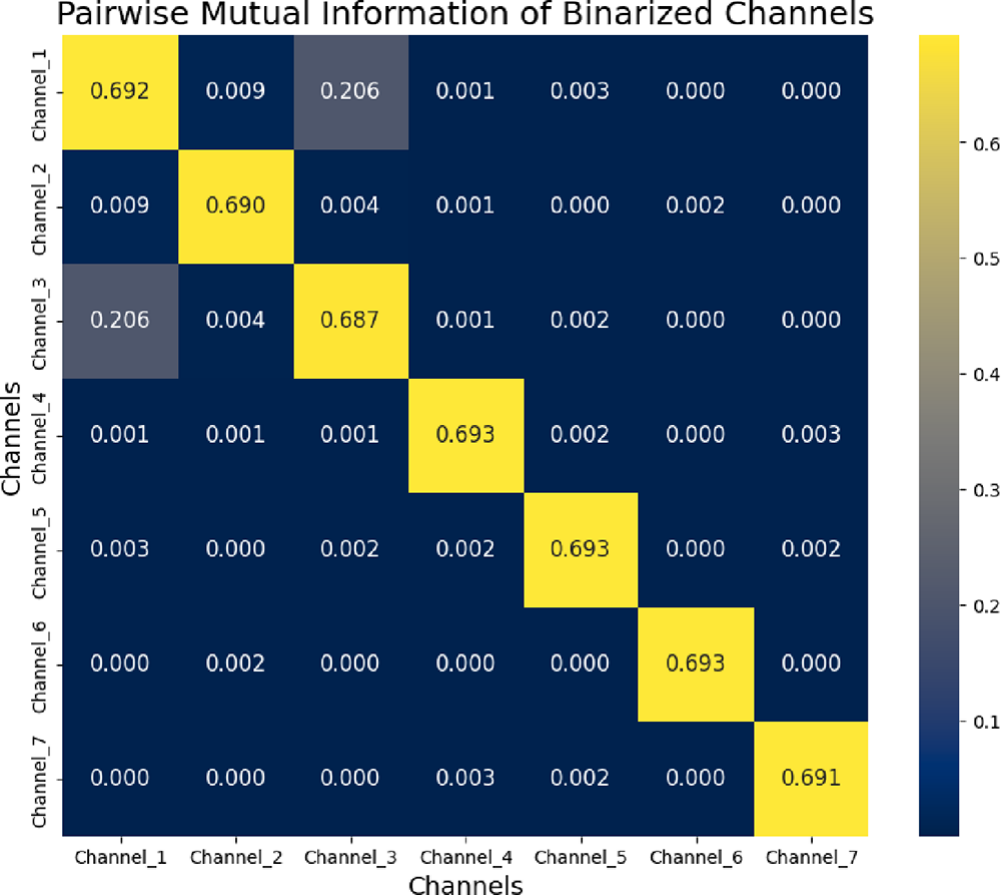
Pairwise mutual information between binarized electrode channels quantifies how much one channel’s state predicts another. Self-information values on the diagonal (∼0.69 bits) indicate that each channel alternates between active and inactive states without being dominated by one mode. Off-diagonal values are uniformly low (0–0.206 bits), showing minimal statistical dependence across all 21 channel pairs. Even the highest value (*I* = 0.206 bits between Channels 1 and 3) represents only a small fraction of the 1-bit maximum, indicating weak predictive relationships. Most pairs exhibit near-zero mutual information, confirming that electrode signals behave largely independently despite occupying the same fermentation medium. This resolves the discrepancy with moderate Pearson correlations, since correlation captures linear relationships in continuous signals, whereas mutual information detects all statistical dependencies in binary data. The overall pattern supports the interpretation that 10 mm electrode spacing preserves local metabolic independence, consistent with the high XOR-entropy measurements. Minor asymmetry around the diagonal reflects finite-sample numerical precision rather than biological effects.

**Table 3:** Summary of Information-Theoretic Metrics for Boolean Logic Gates and Channel Relationships in Grape Must Fermentation System^a^ ^,^ ^b^

**metric**	**mean**	**std dev**	**min**	**max**
gate entropy (bits) across 21 channel pairs				
AND entropy	0.714	0.042	0.560	0.880
OR entropy	0.800	0.038	0.766	0.881
XOR entropy	0.932	0.015	0.826	1.000
NAND entropy	0.714	0.042	0.560	0.880
NOR entropy	0.800	0.038	0.766	0.881
XNOR entropy	0.932	0.015	0.826	1.000
channel self-information and mutual information (bits)				
self-information *H*(*A*)	0.691	0.003	0.687	0.693
mutual information *I*(*A*;*B*)	0.012	0.035	0.000	0.206
coefficient of variation (dimensionless)				
CV_AND_	0.059			
CV_XOR_	0.016			

a Entropy calculated using Shannon formula: *H* = −∑_*i*_ *p*_*i*_ log_2_(*p*_*i*_). Mutual information: *I*(*A*;*B*) = *H*(*A*) + *H*(*B*) – *H*(*A*,*B*). Self-information values near 0.69 bits indicate balanced active/inactive states. XOR entropy consistently approaches maximum (1 bit), confirming temporal asynchrony. Low mutual information (*I* < 0.21 bits for all pairs) demonstrates spatial independence. Coefficient of variation: CV = σ_H_/μ_H_, quantifying interpair variability.

b Shannon entropy values quantify output predictability (0 bits = deterministic, 1 bit = maximally random), while mutual information measures statistical dependence between channel pairs.

#### Principal Component Analysis of Binary Channel States

Principal component analysis (PCA) of binarized electrode states shows a clear breakdown of activation patterns. This reveals how bioelectrochemical processes are organized in time and space during grape must fermentation. You can find more details in Table 4. PCA transforms the original seven-dimensional binary state space using an orthogonal basis, where components are ordered by the amount of variance they explain. For a data matrix $X\in R^{n\times 7}$ with *n* time points and 7 channels, the covariance matrix is defined as

$$C=\frac{1}{n-1}X^{T}X$$ 13

**Table 4:** Principal Component Loadings for Binarized Electrode Channel States in the Grape Must Fermentation System^a^

**channel**	**PC1**	**PC2**	**PC3**	**PC4**	**PC5**	**PC6**	**PC7**
Channel_1	0.436007	0.590285	–0.387043	–0.190406	–0.371915	–0.056505	–0.365911
Channel_2	0.458740	0.078783	0.003208	–0.417061	0.251619	–0.295668	0.677251
Channel_3	0.264271	–0.006825	–0.399364	0.514812	–0.039314	0.580495	0.408744
Channel_4	0.330751	0.382068	0.719419	0.127555	0.262502	0.350558	–0.137823
Channel_5	0.079189	0.182998	–0.204983	0.565207	0.543087	–0.528014	–0.158182
Channel_6	0.460206	–0.563068	–0.190066	–0.241605	0.372168	0.204563	–0.443071
Channel_7	0.449622	–0.385747	0.308203	0.361486	–0.542642	–0.358995	0.006348

a Each entry represents the contribution (loading) of a specific channel to the corresponding principal component, with values ranging from −1 to +1. Higher absolute values indicate stronger contributions to that component. PC1 shows the main mode of synchronized activation across channels, explaining 34.0% of the total variance. Channels 2, 6, and 7 have strong positive loadings (0.459, 0.460, and 0.450), indicating a coordinated activation pattern across these electrodes. PC2 shows a strong positive loading from Channel 1 (0.590), while Channels 6 and 7 have negative loadings (−0.563 and −0.386), representing an anticorrelated activation mode. PC3 through PC7 capture progressively finer variance structures. For example, PC3 (explaining 14.4% of the variance) shows a dominant positive contribution from Channel 4 (0.719). The loading patterns demonstrate that no single channel controls all components, meaning that information is distributed across the electrode array. Channels with similar loadings on different principal components likely share metabolic links or are physically close within the fermentation medium.

PCA finds eigenvectors **v**_*i*_ of **C**. The projection of the data onto each eigenvector is given by

$$z_{i}=Xv_{i}$$ 14

which maximizes variance while keeping the vectors orthogonal.

The first principal component (PC1) shows balanced positive loadings across most channels. Channels 2, 6, and 7 have the strongest contributions at 0.459, 0.460, and 0.450, respectively, while Channel 5 has a minimal contribution of 0.079. This pattern shows that PC1 represents a “global activation mode,” where almost all electrodes change together but at different levels. PC1 represents the main source of variability, expressed as

$$\sigma_{1}^{2}=\lambda_{1}$$ 15

where λ_1_ is the largest eigenvalue of **C**. This eigenvalue reflects the key fluctuations that drive system behavior. Channel 5 shows low PC1 loading but a strong PC5 contribution of 0.543, indicating that this electrode samples a different metabolic process and operates independently from the main collective mode.

The second principal component (PC2) shows a different loading structure. It has a strong positive weight on Channel 1 (0.590), while Channels 6 and 7 show negative loadings (−0.563, −0.386). You can see this in Table 4. This bipolar pattern represents an anticorrelated activation mode, which can be approximated as

$$\mathrm{PC}2\approx 0.59\cdot B_{1}-0.56\cdot B_{6}-0.39\cdot B_{7}+\mathrm{smaller}\;\mathrm{terms}$$ 16

where *B*_*i*_ denotes the binary state of channel *i*.

This suggests that competing metabolic processes may be present: when Channel 1 is active, Channels 6 and 7 tend to be inactive, and vice versa. Such anticorrelation could arise from two main factors: (1) substrate competition, where active fermentation in one area reduces nutrient availability in others; and (2) electrical field effects, where ionic currents near one electrode create potential gradients that influence measurements at other locations.

The variance captured by PC2 (20.1%) is substantially lower than that of PC1 (34.0*%*), indicating that this anticorrelated mode represents a secondary dynamical feature. The orthogonality constraint

$$v_{1}\cdot v_{2}=0$$ 17

ensures that PC2 captures variance independent of the global activation mode represented by PC1. However, orthogonality in the mathematical sense does not guarantee biological independence; the components may still represent interconnected parts of the same metabolic networks.

Figure 10 shows that the system exhibits multiple components. The cumulative variance function is defined as

$$V_{\mathrm{cum}}\left(k\right)=\frac{\overset{k}{\underset{i=1}{\sum }}\lambda_{i}}{\overset{7}{\underset{i=1}{\sum }}\lambda_{i}}$$ 18

which measures the fraction of total variance explained by the first *k* components. The curve saturates slowly: it takes three components to exceed 70% variance and six components to surpass 95%. This indicates that the interchannel relationships are quite complex.

**Figure 10:**
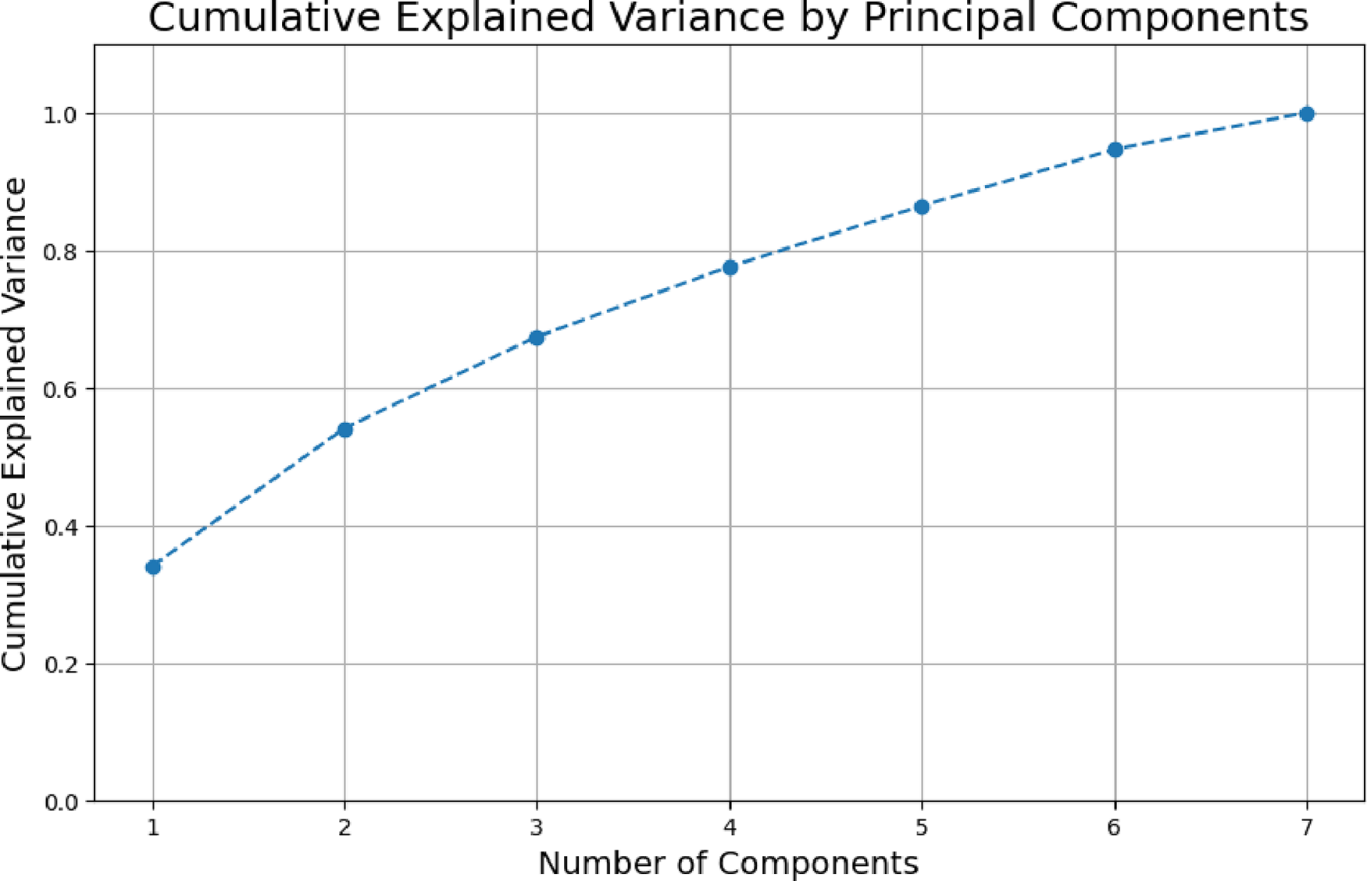
Cumulative explained variance as a function of principal component count for binarized channel states in grape must fermentation system. The first principal component captures 34.0% of total variance, representing the dominant mode of collective channel activation. The first two components make up 54.1% of the variance. This means that about half of the system’s behavioral diversity can be explained by these two activation patterns. By the fourth component, cumulative variance hits 77.6%. This means the seven-channel system mainly relies on 3–4 key coactivation modes, not fully independent channel dynamics. The approach to 100% variance shows that later principal components (PCs) capture smaller parts of system behavior. They highlight subtle or rare activation patterns. The slow rise of the cumulative variance curve shows that it takes 6 components to exceed 95% variance. This points to a complex web of interchannel relationships, where many independent modes play a key role in the system’s dynamics. This structure shows that the fermentation system does not rely on one main oscillator. Instead, it uses multiple, partly independent electrochemical processes. These processes create complex patterns over time and space.

For comparison, a system with a single dominant oscillator would show rapid variance saturation,^66^ reaching *V*_cum_(1) ≈ 0.9. In contrast, completely independent channels would grow linearly, following^67^ *V*_cum_(*k*) ≈ *k*/7. The observed behavior occupies an intermediate regime, suggesting partial coupling among channels. The system’s effective dimensionality can be quantified using the participation ratio:

$$D_{\mathrm{eff}}=\frac{{\left(\overset{7}{\underset{i=1}{\sum }}\lambda_{i}\right)}^{2}}{\overset{7}{\underset{i=1}{\sum }}\lambda_{i}^{2}}$$ 19

For evenly distributed variance (λ_*i*_ = const), this equals 7. If one mode dominates, it approaches 1. The spatial distribution of oscillation frequencies across the seven channels exhibits moderate variance. Calculating from the mean frequencies in Table 1: mean = 0.00122 Hz, variance = 3.56 × 10^–7^ Hz^2^, standard deviation = 5.97 × 10^–4^ Hz, corresponding to a coefficient of variation (CV = SD/mean) of 0.49. This indicates moderate heterogeneity, with frequencies spanning approximately a 5-fold range from the slowest channel (Channel 7:0.00044 Hz) to the fastest (Channel 1:0.00215 Hz). This finding challenges a simple view of fermentation as a uniform process and instead suggests a landscape of partly independent metabolic microenvironments, connected by substrate diffusion and possibly electrical interactions.

The state space visualization in Figure 11 shows the fermentation system’s trajectory in the two-dimensional subspace formed by {**v**_1_,**v**_2_}. Each point (*z*_1_(*t*),*z*_2_(*t*)) represents the system’s configuration at time *t*, projected onto the first two principal components using

$$z_{1}\left(t\right)=\overset{7}{\underset{i=1}{\sum }}v_{1,i}B_{i}\left(t\right)$$ 20

$$z_{2}\left(t\right)=\overset{7}{\underset{i=1}{\sum }}v_{2,i}B_{i}\left(t\right)$$ 21

where *v*_*j*,*i*_ denotes the loading of channel *i* on component *j* (see Table 4). This projection does not exhibit clear clustering, unlike systems with well-defined attractor states. For example, neural networks can form specific memory patterns, and chemical oscillators often display limit cycles. The nearly even distribution of points indicates that exploration of the state space is largely diffusive, a behavior typical of random biological processes. Subtle structure appears in the concentration of points near (*z*_1_, *z*_2_) ≈ (0.5, 0.5), suggesting that most channels with positive PC1 loadings are active, Channel 1 is moderately active, and Channels 6 and 7 show intermediate activity. The range of approximately 2.5 units in both PC1 and PC2 indicates that the system explores almost all possible projections in this two-dimensional space. This means that while some configurations may be preferred, the fermentation dynamics are not confined to a narrow region of the state space. Such broad exploration likely reflects the inherently noisy nature of biological electrochemical processes: thermal fluctuations, random chemical reactions, and turbulent flow prevent the system from settling into regular, low-dimensional patterns.

**Figure 11:**
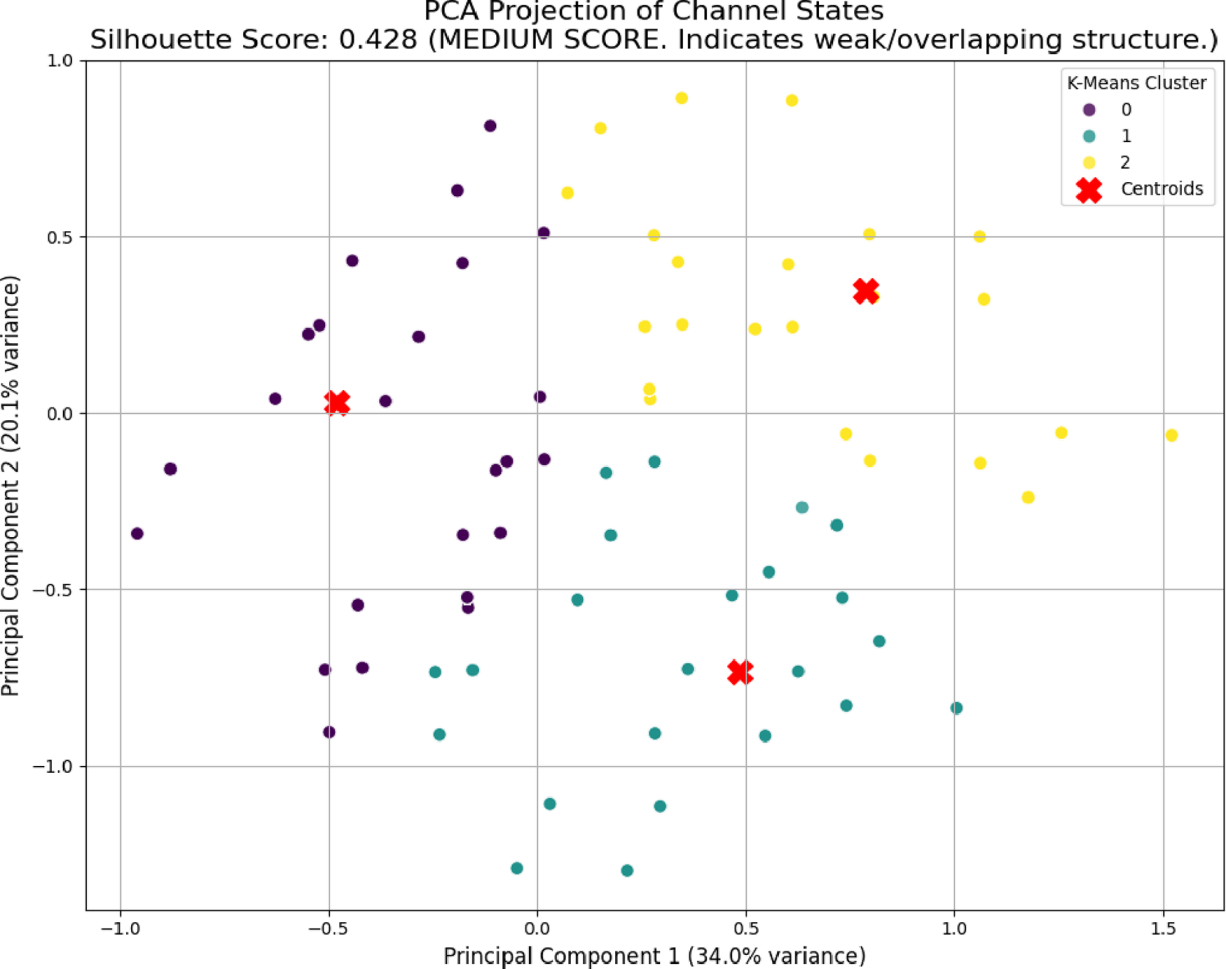
State space projection of binarized channel states onto the first two principal components during grape must fermentation. Each point represents the system configuration at a single time point, with coordinates derived from PC1 and PC2 loadings. The broad scatter distribution and relatively uniform point density indicate extensive state space exploration typical of stochastic biological processes rather than deterministic limit cycles. K-Means clustering (*k* = 3) identifies three centroids (red crosses) with a Silhouette Score of 0.428, indicating weak cluster separation and fluid boundaries between metastable states. The lack of sharp boundaries or voids suggests smooth transitions between activation modes. This compressed representation of the 128 possible binary configurations (2^7^ states) reveals that dynamics concentrate in specific regions defined by the principal components while maintaining broad accessibility across the state space.

Evaluating the PCA framework for binary fermentation data shows both its strengths and weaknesses. The method reduces the 128-dimensional binary state space (2^7^ = 128 possible configurations) to a simpler form. This new representation reveals statistical patterns, but it does lose some information. The variance metric used to rank component importance is

$$\mathrm{Var}\left(z_{i}\right)=\lambda_{i}$$ 22

which works well for continuous data. However, it can be less effective for binary variables, where standard deviation does not clearly represent typical deviations from the mean. Alternative approaches, such as independent component analysis (ICA), aim to extract statistically independent components rather than merely uncorrelated ones, which could uncover new organizational principles. The linear projection

z=Xv 23

cannot capture nonlinear relationships between channels. For example, operations like logical AND or XOR between channel pairs will not appear in the principal component loadings. The original time series preserves temporal dynamics (see Figure S3). In the PCA projection plot, these dynamics are lost because each time point is treated independently, ignoring temporal order. A better analysis might use time-lagged embedding or dynamic mode decomposition, which can reveal time-dependent structures. Despite these limitations, PCA provides valuable insights. It shows that no single channel dominates system behavior, as indicated by the distributed loadings. It also highlights anticorrelated activation patterns and measures the effective dimensionality of the collective dynamics. These findings complement earlier peak-detection and spectral analyses, shifting the focus from individual channel properties to the overall collective behavior of the fermentation system.

### Interpretation and Limitations of Electrical Measurements

Our electrical measurements establish several key findings about grape must fermentation dynamics. Oscillations arise from biological activity, as definitively demonstrated by sterile controls that exhibit drift but lack periodic features. The fermentation dynamics exhibit substantial spatial heterogeneity, reflected in consistently low interchannel correlations. Environmental temperature modulates metabolic activity, with correlations ranging between 0.245 and 0.585 (Figure 5). The system displays brown-noise temporal structure, suggesting integration of stochastic metabolic fluctuations over extended time scales. Spatial coupling between regions produces behavior analogous to Boolean logic operations, with XOR gates achieving near-maximal entropy (Table 3). However, several fundamental questions remain unresolved without parallel metabolic characterization. The specific metabolic pathways responsible for generating voltage oscillations remain unidentified. The quantitative link between electrical signal amplitude and metabolic flux is unknown. The relative contributions of pH changes, redox processes, and ionic transport to the measured signals cannot be distinguished with electrical measurements alone. Relationships between yeast cell density and oscillation characteristics (frequency, amplitude) are not determined. Substrate depletion kinetics across different spatial regions are not measured. Patterns of metabolite accumulation and their electrical signatures remain uncharacterized.

These limitations necessitate future work integrating electrical monitoring with metabolic measurements. We are planning follow-up studies incorporating real-time pH and dissolved oxygen sensing, periodic metabolite profiling via HPLC/GC-MS, microbial enumeration and spatial distribution analysis, and controlled perturbation experiments. Such integrated approaches will enable quantitative validation of our bioelectrochemical interpretations and establish whether electrical monitoring can serve as a practical proxy for fermentation state assessment.

Our results are specific to Roditis grape must from the Patras region prepared by traditional methods. Different grape varieties (with varying sugar, acid, and phenolic content), geographic origins (with distinct native microbiota), harvest timing (affecting ripeness and microbial load), or preparation methods would likely produce quantitatively different oscillation characteristics—potentially different frequencies, amplitudes, spatial patterns, or environmental sensitivities. However, we hypothesize that the qualitative phenomena we observe—spontaneous bioelectrochemical oscillations, spatial heterogeneity, brown noise temporal structure, and environmental coupling—represent general features of natural fermentation systems rather than Roditis-specific properties. Systematic comparative studies across varieties, regions, and preparation methods are needed to distinguish universal fermentation dynamics from substrate-specific behaviors.

The peak detection parameters (4.0 mV threshold, 400 s minimum separation) represent operational choices that balance sensitivity against specificity. Different parameter values lead to varied peak counts. Lower thresholds or shorter separations find more events but raise false positives. In contrast, higher thresholds or longer separations boost confidence but may miss low-amplitude or quick events. The parameters we used fit the main oscillation time scales in our system (465–2271 s). Yet, they might not apply to fermentation systems with different frequencies.

## Conclusions

This study is the first to fully describe spontaneous bioelectrochemical oscillations in grape must fermentation. It shows a complex pattern of spatiotemporal dynamics. This challenges the common idea that fermentation is a uniform process. Our multichannel electrode array found oscillatory patterns at ultralow frequencies (0.00044–0.00215 Hz). These frequencies correspond to periods of 7.8–37.8 min and match known metabolic cycles in yeast populations. The spectral analysis revealed brown noise characteristics (γ > 2) rather than the white or pink noise typically assumed in biological systems. This finding is important for fermentation control. The strong time correlations and memory effects show that the system remembers past states through several oscillation cycles. Substrate depletion, metabolite buildup, and pH changes have lasting effects. Conventional control strategies, which assume white noise, do not address these issues. Environmental sensitivity analysis showed that temperature is the main external factor affecting oscillatory behavior. In contrast, humidity decreases signal amplitude. These correlations offer useful guidelines for improving fermentation conditions. However, the brown noise traits indicate that quick environmental changes might have delayed and lasting impacts on the system. The spatial and temporal relationships between electrode measurements can be formally characterized using Boolean logic frameworks, revealing that interelectrode coupling produces patterns analogous to logic gate operations. XOR operations have high entropy at 0.93 bits. They also show low mutual information between channels, less than 0.206 bits. This means the system stays locally independent but still shows global organization. This duality—consistent timing differences with varied spatial activity—shows that fermentation systems process information through metabolic networks. Self-organized criticality signatures show up in grape must fermentation. This includes power-law scaling and multiscale temporal correlations. These features link fermentation to complex adaptive systems. The system operates near a critical point where local metabolic processes generate emergent collective behaviors without central control. This finding connects fermentation science with complexity theory. It suggests new ways to optimize processes. Instead of using external control, focus on controlling criticality. Our results set the stage for future work: The brown noise framework predicts variance growth and stability in fermentation systems. The Boolean logic implementation opens doors for biological computing with fermentation substrates. The spatial maps highlight targets for optimizing fermentation. The environmental patterns help create models for batch consistency. These findings extend beyond fermentation science to inform our understanding of bioelectrochemical systems generally. Traditional food preparation methods can create complex behaviors. This suggests that biological systems might use information processing methods that are very different from those in engineered systems. These systems do not store information as separate states. Instead, they use statistical patterns from group configurations and how these patterns change over time. In conclusion, grape must fermentation is more than just a biochemical change. It acts like a bioelectrochemical computer. It processes information using metabolic networks. It also stays strong through brown noise dynamics and self-organized criticality. This view creates new paths for basic research in biological information processing. It also has practical uses in fermentation monitoring, control, and optimization.

## Supplementary Material



## Notes

Table S1: detailed Boolean logic gate statistics for seven representative channel pairs showing high/low state counts, activation percentages, transition rates, and Shannon entropy values; Figure S1: classification framework for stochastic noise types (white, pink, brown, autoregressive) illustrating power spectral density characteristics and their relevance to fermentation dynamics; Figure S2: time-series environmental monitoring data (temperature, humidity, dew point) spanning the experimental period (July 8–10, 2025); Figure S3: binarized voltage states for all seven electrode channels showing temporal activation patterns and switching dynamics using a 2.0 mV threshold; and Figure S4: representative Boolean logic gate operations (AND, OR, XOR, NAND, NOR, XNOR) applied to Channel 1 and Channel 2 binary states, demonstrating the analytical framework for interchannel computational relationships (PDF)
