# Supplementary material for: Grape Must as a Bioelectrochemical Processor
Source: ACS Omega. 2026 Feb 17;11(8):13473–95. doi: 10.1021/acsomega.5c10998 (PMC12961455; doi:10.1021/acsomega.5c10998)
Supplement: Supplementary file 1 [file ao5c10998_si_001.pdf]

Supporting Information

Grape Must as a Bioelectrochemical Processor

Panagiotis Mougkogiannis[1,*] and Andrew Adamatzky[1]

[1]Unconventional Computing Laboratory, University of the West of England, Bristol, UK, BS16 1QY

Email: Panagiotis.Mougkogiannis@uwe.ac.uk

Table S1 shows Boolean gate statistics for representative channel pairs. Figure S1 illustrates the noise classification framework. Figure S2 presents environmental monitoring data. Figure S3 displays binarized voltage states for all channels. Figure S4 demonstrates Boolean logic operations for a representative channel pair.

Classifying stochastic processes by their spectral traits gives us key insights. This helps us understand the time-related patterns in bioelectrochemical oscillations. Different noise types, shown in in the Supporting Information (Figure S1), range from uncorrelated white noise to strongly integrated brown noise. These types represent distinct dynamical regimes in fermentation systems. Figure S1 shows how we classify stochastic processes. This classification is based on their spectral traits and how their values relate over time. The visualization shows power spectral density per octave as a function of temporal frequency. It reveals how various noise types affect different timescales.

White noise ($\gamma = 0$) has a flat power spectral density and no time correlations. This makes it emphasize short timescales when viewed per octave.

Pink noise or **$1/f$ noise** ($\gamma = 1$) has a special scale-invariant property. It spreads power evenly across all time scales, from daily to centennial. This makes it ideal for modeling environmental and biological processes that operate over different timescales.

Brown noise ($\gamma = 2$, $S(f) \propto 1/f^2$) represents a random walk process with unbounded variance growth and strong emphasis on long timescales.

Autoregressive noise differs from power-law noise types. It shows exponential decay in its correlation structure over a characteristic timescale τ, concentrating power within a limited frequency band.

The spectral slope parameter γ in the relationship

$$S(f) \propto \frac{1}{f^\gamma} \tag{1}$$

measures the strength of temporal correlation.

At $\gamma = 0$, fluctuations are uncorrelated. For $0 < \gamma < 2$, there are intermediate memory effects. At $\gamma = 2$, the process represents pure integration. This framework, originally developed for geophysical time series, helps us understand our fermentation system.

Table S1: Boolean logic gate statistics for selected channel pairs in grape must fermentation system. These seven pairs were chosen to represent the range of observed behaviors: high entropy pairs (Channels 1&5, 2&4), low entropy pairs (Channel 6&7), high co-activation (Channel 4&5), and low co-activation (Channel 1&2). Binary states were derived using a 2.0 mV threshold, with gate operations performed on all unique electrode pair combinations. High Count indicates the number of time samples (out of 198,000 total) where the gate output equaled 1 (active state); Low Count indicates samples where output equaled 0 (inactive state). A high percentage shows how often the gate output is 1. The transition rate measures how often the state changes. Entropy indicates how random the output is, with a maximum of 1 bit when 0 and 1 are equally likely.

Channel Pair	Gate	High Count	Low Count	High %	Trans. Rate	Entropy
Channel 1 & 2	AND	36 584	161 482	18.47	0.000 51	0.690 26
	OR	91 746	106 320	46.32	0.001 74	0.996 09
	XOR	55 162	142 904	27.85	0.002 25	0.853 40
Channel 1 & 4	AND	70 954	127 112	35.82	0.001 51	0.941 21
	OR	150 453	47 613	75.96	0.001 61	0.795 69
	XOR	79 499	118 567	40.14	0.003 12	0.971 75
Channel 1 & 5	AND	75 859	122 207	38.30	0.001 37	0.960 13
	OR	174 276	23 790	87.99	0.001 86	0.529 68
	XOR	98 417	99 649	49.69	0.003 22	0.999 97
Channel 2 & 4	AND	43 425	154 641	21.92	0.000 57	0.758 79
	OR	139 390	58 676	70.38	0.001 48	0.876 65
	XOR	95 965	102 101	48.45	0.002 04	0.999 31
Channel 4 & 5	AND	118 587	79 479	59.87	0.002 26	0.971 69
	OR	186 033	12 033	93.92	0.000 77	0.330 42
	XOR	67 446	130 620	34.05	0.003 02	0.925 32
Channel 5 & 6	AND	62 875	135 191	31.74	0.003 20	0.901 58
	OR	179 901	18 165	90.83	0.001 24	0.442 16
	XOR	117 026	81 040	59.08	0.004 43	0.976 06
Channel 6 & 7	AND	44 961	153 105	22.70	0.001 22	0.772 75
	OR	96 403	101 663	48.67	0.003 17	0.999 49
	XOR	51 442	146 624	25.97	0.004 38	0.826 33

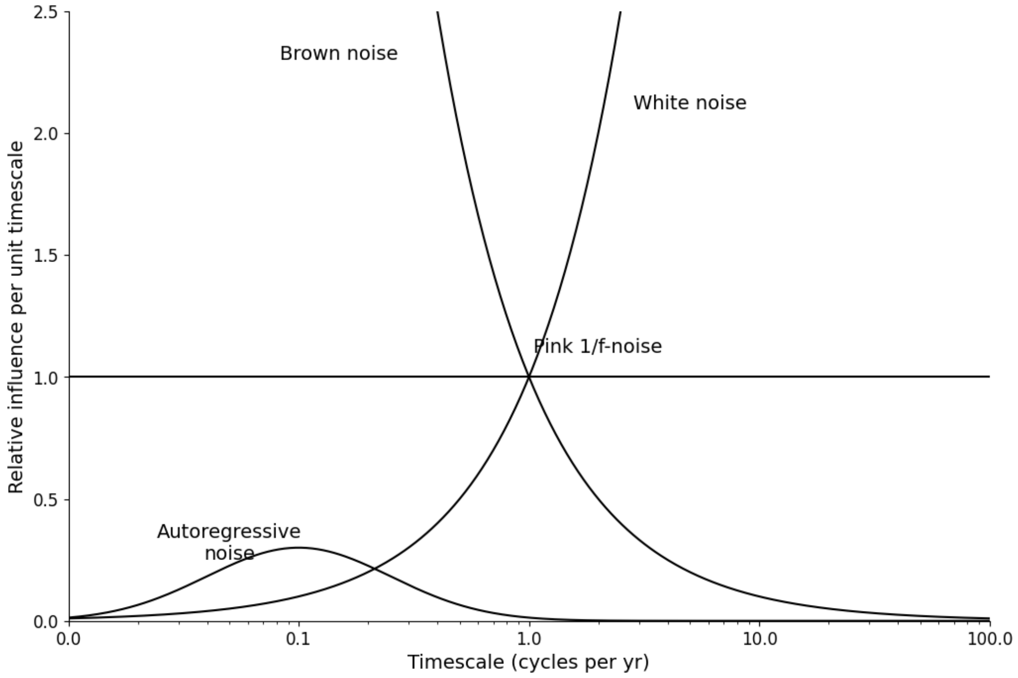

Figure S1: Classifying stochastic processes by their spectral traits and time correlation structures shows the broad range of $1/f$-noise processes. These are important for understanding biological and environmental systems. The figure shows how different noise types spread power across timescales. It illustrates relative influence per unit timescale (spectral density per octave) as a function of temporal frequency. **White noise** ($\gamma = 0$, flat power spectral density $S(f) \propto f^0$) shows no time correlation. It highlights short timescales too much when looked at per octave. This makes it unsuitable for modeling environmental processes that involve multiple time scales. **Pink or $1/f$-noise** ($\gamma = 1$, $S(f) \propto 1/f$) is unique in this group. It spreads influence evenly across all time scales. This means it gives equal importance to daily, yearly, decadal, and centennial fluctuations. Pink noise is a natural model for environmental changes. It works well because it doesn't have the short-timescale bias of white noise or the long-timescale dominance of brown noise. This scale-invariant property helps it represent various processes happening over different timescales. **Brown noise** ($\gamma = 2$, $S(f) \propto 1/f^2$, also known as Brownian noise or random walk) shows non-stationary behavior. The variable drifts endlessly, focusing on long timescales. Its variance grows linearly with the length of observation. **Autoregressive noise** has a correlation structure that decreases quickly over time. This decay happens at a characteristic timescale, τ. It focuses power within a narrow frequency band and does not show the power-law scaling found in natural environmental processes. The spectral slope γ in the relationship $S(f) \propto 1/f^\gamma$ quantifies the degree of temporal correlation: $\gamma = 0$ indicates uncorrelated fluctuations (white noise), $0 < \gamma < 2$ represents intermediate correlation structures with memory effects extending across multiple timescales, and $\gamma = 2$ corresponds to pure integration (random walk). In grape must fermentation systems, the brown noise characteristics show spectral slopes between -2.01 and -3.28 (see Figure **??**). This suggests strong temporal integration and diffusion-limited dynamics. Here, substrate depletion, metabolite buildup, and pH changes have lasting effects that influence several oscillation cycles. This classification framework started with geophysical time series and later applied to ecological dynamics. It offers a key foundation for understanding the time-related patterns in bioelectrochemical oscillations. It helps to separate external noise from environmental factors and internal noise from metabolic processes.[?]

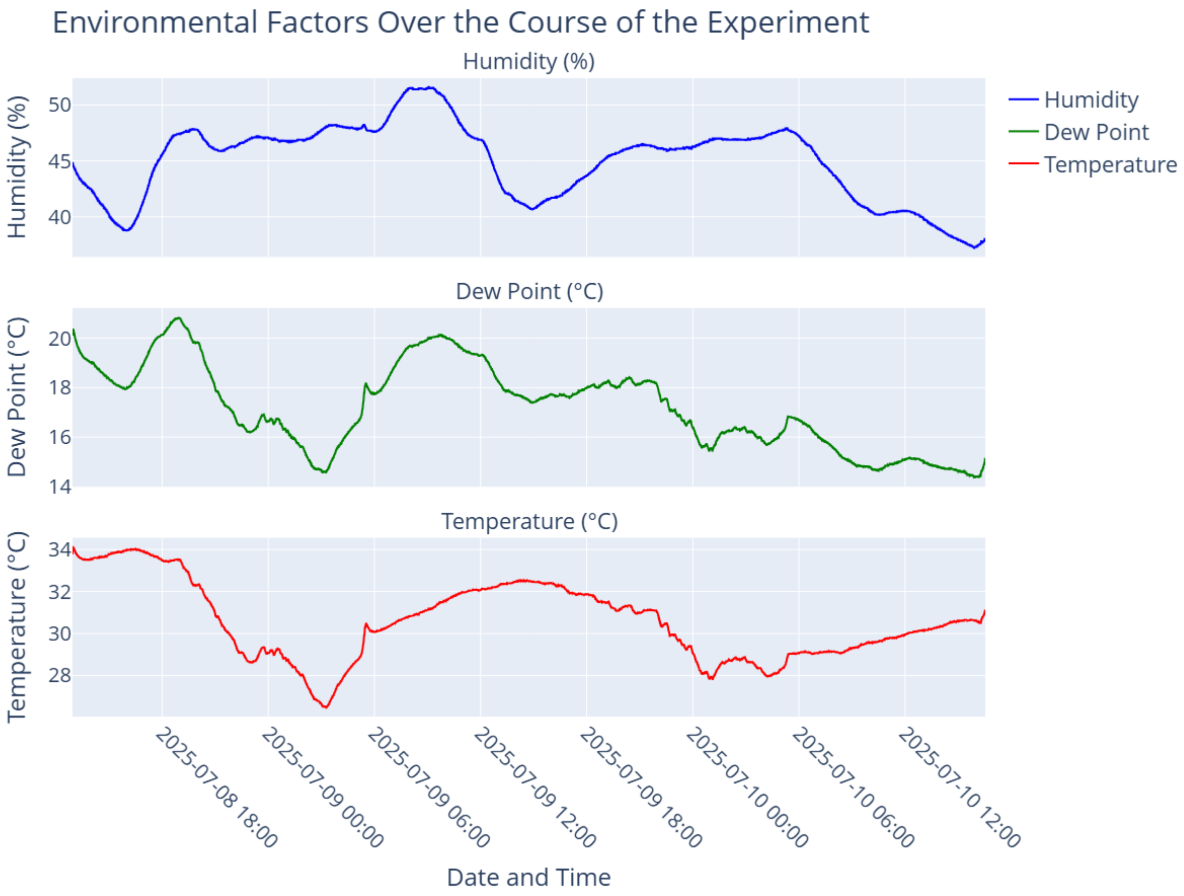

Figure S2: Time series plots of environmental factors over the course of the experiment, spanning from July 8, 2025, to July 10, 2025. Temperature (blue) and humidity (orange) are the independent environmental variables measured; dew point (green) is shown for completeness but is mathematically derived from temperature and humidity rather than representing an independent factor. The top subplot shows humidity (%) fluctuating between about 40% and 50%. It has regular peaks and dips, likely affected by daily cycles or lab conditions. The middle subplot depicts dew point (°C) varying from 14 °C to 20 °C, exhibiting similar oscillatory patterns that may correlate with humidity changes. The bottom subplot illustrates temperature (°C) decreasing overall from 34 °C to around 28 °C, with notable dips and rises that could impact the mustalevria oscillations. These trends show changing environmental conditions during the experiment, so it is important to consider these variations when analyzing correlations with channel data.

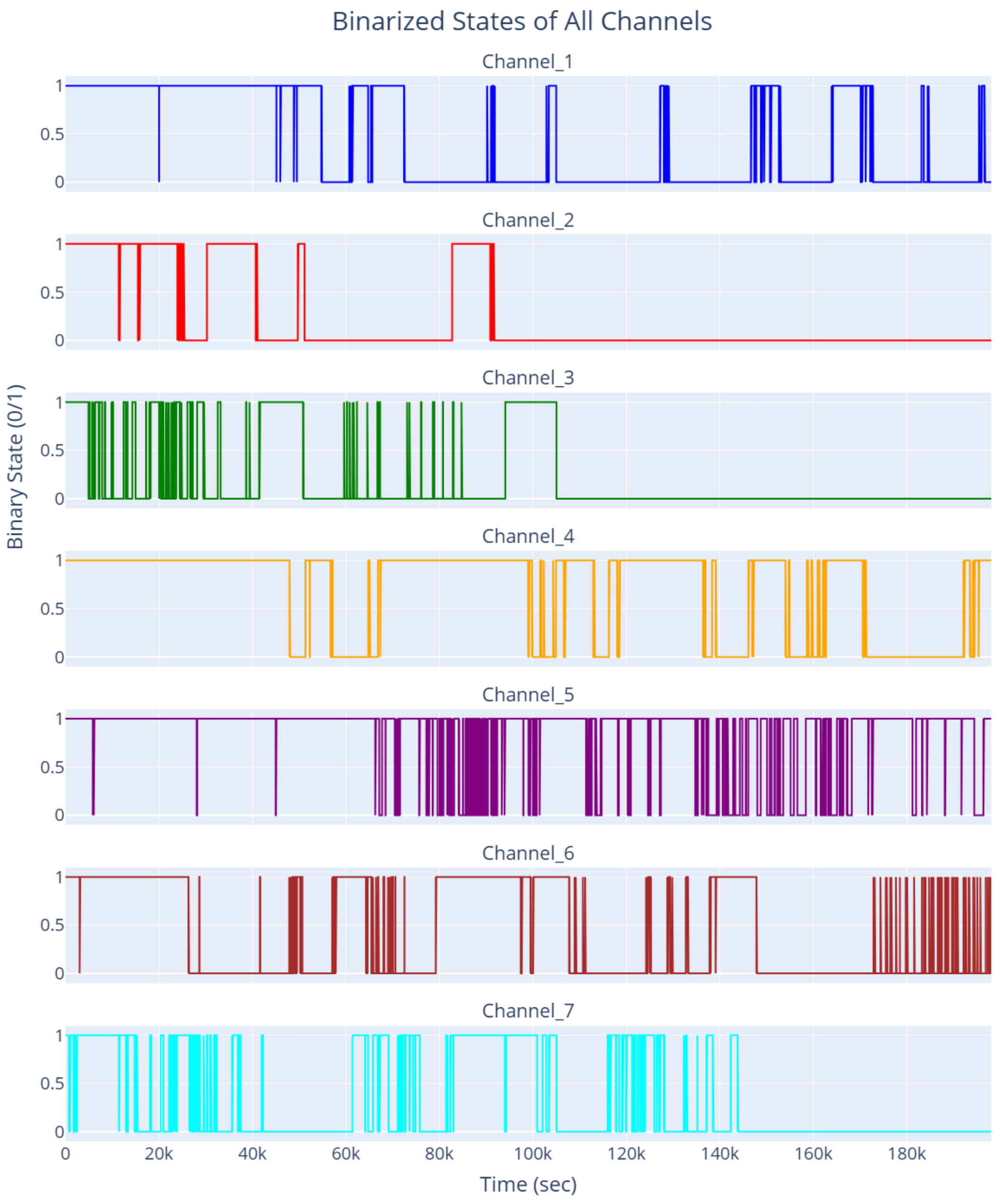

Figure S3: Binarized voltage states for all seven differential electrode channels in the grape must fermentation system during the 200,000-second measurement period. Each channel's voltage signal is converted into binary states using a 2.0 mV threshold. Values above this threshold become state 1 (active/high), while values below it become state 0 (inactive/low). The temporal patterns show different activation dynamics across channels: Channel 1 has sporadic bursts and long quiet periods. Channel 2 starts with activity but then goes quiet for a while. Channel 3 features high-frequency switching with dense pulses at first. Channel 4 alternates between active and inactive times with medium pulse widths. Channel 5 stays active the longest and has frequent changes. Channel 6 has a bimodal pattern with bursts in the early and late phases. Channel 7 shows complex and irregular switching. These binarized representations enable Boolean logic operations and information-theoretic analysis of inter-channel relationships.

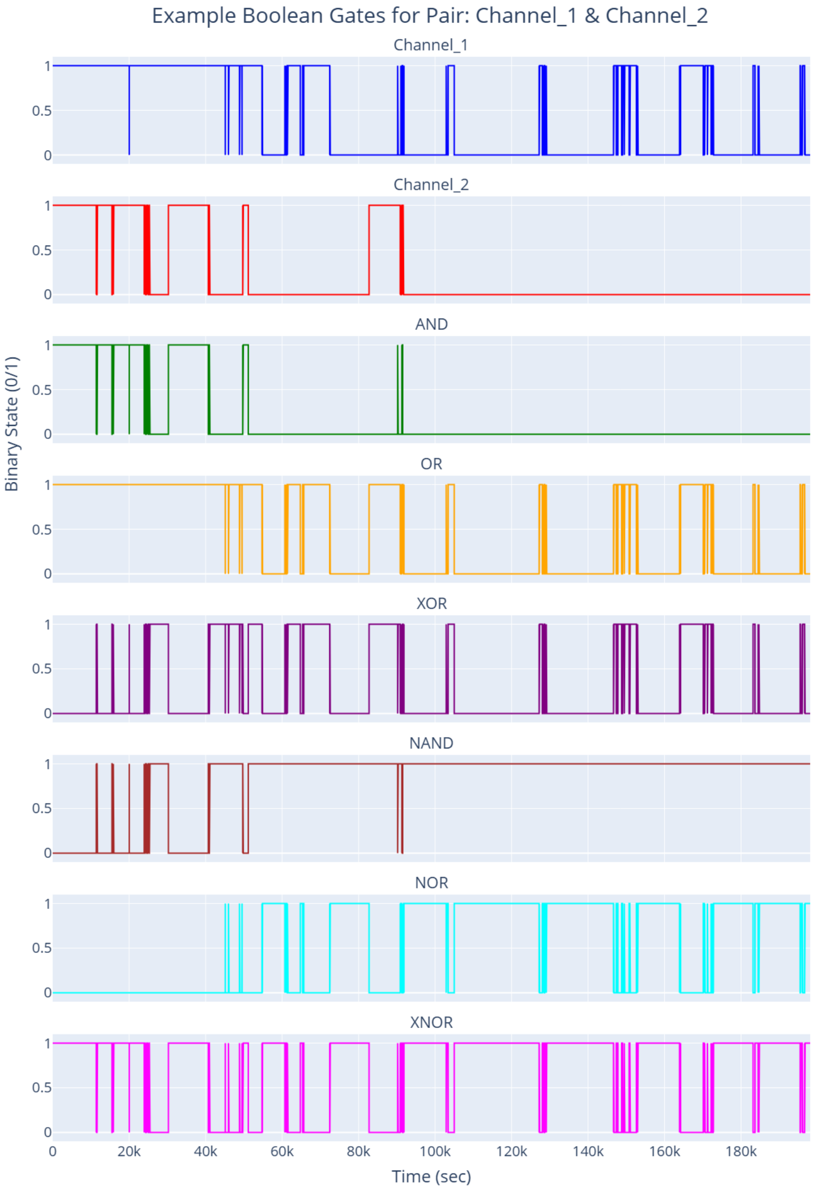

Figure S4: Example Boolean logic gate operations applied to Channel 1 and Channel 2 binary states, shown as a representative case to illustrate the analytical framework. The first two panels show the input signals from each channel. The next six panels display the outputs of basic logic gates: **AND**: active only when both inputs are high. **OR**: active when either input is high. **XOR**: active when inputs differ. **NAND**: inverted AND. **NOR**: inverted OR. **XNOR**: inverted XOR. For this channel pair, the AND gate shows sparse activation, with 18.47% in a high state and an entropy of 0.69 bits. This means high states occur rarely together. In contrast, the OR gate has broader activation, at 46.32% high state and an entropy of 0.996 bits, reflecting activity from either channel. The XOR gate (27.85% high state, entropy 0.853 bits) highlights periods of asynchronous behavior between the two channels. Transition rates range from 0.00051 (AND/NAND) to 0.00225 (XOR/XNOR), indicating the temporal dynamics of logical relationships. These gate operations show how separated electrode pairs perform basic computations through their bioelectrochemical coupling during fermentation.
